# Multiple sclerosis: molecular pathogenesis and therapeutic intervention

**DOI:** 10.1038/s41392-025-02415-4

**Published:** 2025-10-02

**Authors:** Imane Boutitah-Benyaich, Herena Eixarch, Javier Villacieros-Álvarez, Arnau Hervera, Álvaro Cobo-Calvo, Xavier Montalban, Carmen Espejo

**Affiliations:** 1 Servei de Neurologia, Centre d’Esclerosi Múltiple de Catalunya (Cemcat), Vall d’Hebron Institut de Recerca (VHIR), Hospital Universitari Vall d’Hebron, Barcelona, Spain; 2 Universitat Autònoma de Barcelona, Bellaterra, Cerdanyola del Vallès Spain; 3 Centro de Investigación Biomédica en Red de Enfermedades Neurodegenerativas (CIBERNED), Instituto de Salud Carlos III, Ministerio de Ciencia, Innovación y Universidades, Madrid, Spain

**Keywords:** Neurological disorders, Neuroimmunology

## Abstract

Multiple sclerosis is a chronic immune-mediated disorder of the central nervous system characterized by demyelination, axonal loss, and neuroinflammation, culminating in progressive neurological disability. Despite significant advances in understanding its immunopathogenesis, current immunotherapies remain limited in their ability to halt disease progression, making multiple sclerosis incurable and highlighting the critical need for novel therapeutic strategies. Antigen-specific immunotherapy represents a groundbreaking approach that aims to restore immune tolerance to myelin-derived antigens while preserving the protective functions of the immune system. Unlike broad immunosuppressive strategies, antigen-specific immunotherapy offers the potential for highly targeted modulation of pathogenic immune responses, reducing off-target effects and enhancing safety profiles. Over the last two decades, preclinical studies and clinical trials have explored diverse antigen-specific immunotherapy modalities, ranging from peptide-based vaccines to nanoparticle platforms, each aimed at achieving durable tolerance in multiple sclerosis. This review provides a comprehensive overview of multiple sclerosis, covering its etiology, clinical features, pathogenesis, pathology, and current therapeutic approaches. Thus, it delves into the current state of antigen-specific immunotherapy research, critically examining its successes and limitations while addressing the translational challenges that must be overcome to realize its therapeutic potential. By integrating insights from immunology, biotechnology, and translational medicine, we propose directions for advancing antigen-specific approaches in the quest for transformative multiple sclerosis therapies.

## Introduction

Multiple sclerosis (MS) is a chronic immune-mediated disorder of the central nervous system (CNS) characterized by demyelination, axonal loss, and neuroinflammation, ultimately leading to progressive physical and cognitive disabilities.^1^ Indeed, it is the most common neurological condition in young adults^2^ and has a significant impact on social, economic, and individual well-being. Progress in MS therapy over the past years has represented a remarkable achievement in the field of medicine. However, although numerous clinical trials are underway, there is still no curative treatment, and most current therapies are aimed at reducing inflammation in a nonspecific way. These therapies are only effective in the early stages of the disease, when inflammation predominates, but they generally fail to halt disease progression. In addition, when administered long term, such therapies are often associated with adverse effects, which are sometimes severe. Ideally, a therapy should be both safe and well tolerated while specifically eliminating or neutralizing the immune cells responsible for the autoimmune attack on myelin components or antigens without compromising overall immune function. For this reason, the induction of antigen-specific immune tolerance has long been considered the elusive “holy grail” of autoimmune disease treatment, particularly in MS. This review provides a comprehensive overview of MS, covering its etiology, clinical features, pathogenesis, pathology, and current therapeutic approaches. It then explores the transformative potential of antigen-specific immunotherapy in redefining MS management, offering a targeted approach to restore immune tolerance while preserving immune surveillance, presenting a significant advantage over broad immunosuppressive therapies.

## Epidemiology, clinical and pathological features of MS

### Epidemiology

According to the Atlas of MS (www.atlasofms.org), an estimated 2.9 million people worldwide are living with MS, with a global prevalence of 35.9 per 100,000 people. However, its prevalence and incidence vary significantly across regions and are influenced by genetic, environmental, and lifestyle factors. MS prevalence generally increases with increasing distance from the equator, with the European and American regions reporting the highest rates (111–300 cases per 100,000) and the African and Western Pacific regions showing the lowest rates (5 per 100,000).^2–6^ This latitudinal gradient is also observed within individual countries where data are available. For example, prevalence rates are higher in the northern regions of the United States than in the southern regions,^4,7^ among other countries with similar patterns. The incidence of MS, which refers to the number of new cases diagnosed annually, also varies. It is estimated at approximately 2.1 cases per 100,000 people per year; however, this rate varies by region, reflecting prevalence trends. Europe reported the highest incidence at 6.8, followed by the Americas at 4.8. In contrast, the lowest incidence rates are observed in Southeast Asia and Africa, at 0.4.^8^ Overall, evidence suggests that MS incidence genuinely increased from the mid-1950s to the late 20th century; since then, it has stabilized or even declined to the level reported in approximately 1995.^9^ This increase may be attributed to better diagnostic techniques, improved disease awareness, and potential environmental changes. MS is most commonly diagnosed between 20 and 40 years of age. Nevertheless, it can begin either in the first years or in the last decades of life,^10–13^ and women are affected three times as often as men are affected, and this ratio is disproportionately increasing.^14–16^ This trend underscores the importance of the interplay between genetic and environmental factors, which are further explored in the sections on genetic factors and environmental triggers.

### Diagnostic criteria: clinical, imaging, and laboratory tests

Since MS lacks a specific biological marker, its diagnosis relies on the combination of characteristic clinical, laboratory and radiological features incorporated into the so-called McDonald criteria.^17,18^ These criteria have evolved over time, refining definitions in response to advances in understanding the disease and the development of new techniques. Notably, magnetic resonance imaging (MRI) has been instrumental in enabling earlier diagnosis and treatment.^19,20^

In terms of laboratory tests, cerebrospinal fluid (CSF) analysis plays a pivotal role, as the presence of CSF, but not serum, IgG oligoclonal bands (OCBs) is a frequent finding in MS patients. Indeed, this biomarker has been incorporated into the McDonald criteria as an alternative criterion for dissemination in time (DIT) when MRI criteria are not met.^18,21^

Although not yet formally included in the diagnostic criteria, other paraclinical tests, such as visual evoked potentials (VEPs), somatosensory evoked potentials (SSEPs), and optical coherence tomography (OCT), can further support MS diagnosis.^22^ Additionally, it is crucial to emphasize that MS remains a diagnosis of exclusion, making comprehensive evaluation essential for ruling out alternative diseases.^18,23^

In the following sections, we describe in more detail the characteristic clinical, imaging and laboratory findings of MS. Table 1 summarizes the typical and atypical features of MS.

**Table 1:** Typical and atypical (“Red Flags”) characteristics in multiple sclerosis

Feature	Typical MS characteristics	Atypical (“red flags”) characteristics
**Clinical**	Subacute onset Unilateral optic neuritis Incomplete spinal cord syndrome Internuclear ophthalmoplegia	Hyperacute or chronic onset Bilateral severe optic neuritis Complete transverse myelitis Encephalopathy, headache Persistent nausea, vomiting or hiccups Prominent ophthalmoplegia
**MRI**	Oval-shaped lesions Periventricular (Dawson’s fingers) Inferotemporal location Juxtacortical involving U-fibers Partial myelitis Central vein sign Chronic “black holes”, slowly expanding lesions (SELs) Nodular or “open-ring” enhancement	Large tumefactive, ill-defined lesions Longitudinally extensive spinal cord lesions (≥3 vertebral segments) Prominent cortical involvement Leptomeningeal enhancement, persistent enhancement
**CSF**	CSF-restricted oligoclonal bands, elevated KFLC, and elevated IgG index Mild lymphocytic pleocytosis	Marked pleocytosis (> 50 cells/mm^3^), polymorphonuclear predominance Significantly elevated proteins Hypoglycorrhachia Absence or transient CSF-restricted oligoclonal bands

*MS* multiple sclerosis, *MRI* magnetic resonance imaging, *CSF* cerebrospinal fluid, *KFLC* kappa-free light chain

#### Clinical features

MS typically presents with episodes of neurological dysfunction (relapses) in the absence of fever or concomitant infectious events and is characterized by diverse symptoms depending on the location of the demyelinating lesions within the CNS.^24^ The most common clinical manifestations include unilateral visual impairment accompanied by ocular pain (optic neuritis), unilateral sensory or motor deficits with or without sphincter dysfunction (partial myelitis), diplopia, imbalance, or vertigo (infratentorial syndrome).^25^ Occasionally, MS may present as multifocal, simultaneously affecting various CNS regions.^26^ Symptoms typically develop acutely or subacutely, within hours or days, persist for at least 24 hours, and usually resolve over days to weeks. In some patients, symptoms may be paroxysmal—short-lasting (seconds to minutes)—but recurrent over periods exceeding 24 hours.^27^ Objective neurological findings are essential during clinical evaluation to define these episodes as true relapses. Recovery from relapses is frequently partial, resulting in progressive disability accumulation correlated with relapse frequency and severity and potentially transitioning into a secondary progressive phase.^28^

Nonspecific symptoms such as fatigue, mood disturbances, and cognitive dysfunction are common among MS patients but should not be considered relapses if presented in isolation.^29^ Certain atypical presentations or “red flags” that should prompt the consideration of alternative diagnoses include hyperacute or chronic symptom onset, bilateral optic neuritis, severe unilateral optic neuritis with poor visual recovery, complete myelitis featuring bilateral motor or sensory symptoms accompanied by significant sphincter dysfunction, intractable nausea, vomiting, hiccups, encephalopathy, or ophthalmoplegia^30^ (Table 1). It is also important to differentiate true relapses from “pseudorelapses”, which refer to transient worsening of existing neurological symptoms in response to external factors such as infections, fever, or stress, without the presence of new demyelinating lesions. Temporary worsening of symptoms triggered by increases in body temperature (e.g., hot weather, exercise, or fever) is known as Uhthoff’s phenomenon.^31^

#### MRI features

The inclusion of MRI in the McDonald criteria was a milestone in MS diagnosis. In addition to diagnosis, MRI plays a critical role in monitoring disease activity. Typically, MS lesions appear as oval or round hyperintense areas on T2-weighted and fluid-attenuated inversion recovery (FLAIR) sequences in the white matter, generally ranging from 3 mm to several centimeters in diameter, with demarcated margins. The four typical locations that are included in the diagnostic criteria are the periventricular, juxtacortical/cortical, infratentorial, and spinal cord. The inclusion of the optic nerve as the fifth topography to fulfill MS criteria has been proposed by a panel of experts, but it has not yet been published.^22,32^ Some key MRI features have been proposed to be highly suggestive of MS and could aid in the diagnosis of uncertain cases: perpendicular orientation of the main axis of the lesion to the lateral ventricles (“Dawson´s fingers”), lesions located in the inferior temporal lobe, and juxtacortical lesions involving U-fibers.^33,34^ Active inflammatory lesions often show enhancement after gadolinium administration, indicating disruption of the blood‒brain barrier (BBB), which typically resolves within four weeks. The enhancement within the lesion usually presents as nodular or “open-ring” patterns. The presence of leptomeningeal enhancement in postcontrast T1-weighted sequences is extremely infrequent in MS and should raise the suspicion of alternative diagnoses, such as neurosarcoidosis or vasculitis, among others. Other “red flags” include punctate or miliary pattern, purely cortical enhancement, band-like pattern, cloud-like pattern, or patchy and persistent enhancement (Table 1). On the other hand, chronic lesions may appear hypointense in T1-weighted sequences (“black holes”). This sign reflects irreversible neuroaxonal damage and is more frequent in secondary forms of MS.^35^ Recently, a subgroup of these lesions, termed slowly expanding lesions (SELs), has been shown to slowly and progressively increase in size and hypointensity on T1-weighted images and hyperintensity on T2-FLAIR images. This radiological finding corresponds to the chronic active (“smoldering”) plaques described in neuropathological studies, representing lesions characterized by chronic inflammatory activity mediated by macrophages and microglia, along with progressive tissue damage. SELs could explain disability progression independent of relapse activity (PIRA).^36^

Notably, two radiological signs have been proposed as supporting features for the diagnosis of MS because of their high specificity: the central vein sign (CVS) and the presence of paramagnetic rim lesions (PRLs).^32^ CVS is characterized by lesions centered around a small vein detectable on high-resolution MR images. PRLs reflect chronic active lesions with a slowly expanding rim of inflammation, which are associated with ongoing disease activity and progression and can be detected on susceptibility-based MRI sequences.^37,38^

#### Laboratory findings

Laboratory investigations aid in the diagnosis of MS, although no single test is diagnostic by itself, CSF analysis frequently reveals OCBs in the CSF but not in the serum, indicating intrathecal antibody production. While OCBs are present in as many as 90% of MS patients, they are not specific to the disease and can also appear in other conditions, such as neurosarcoidosis, vasculitis, and Behçet disease. An elevated IgG index and mild pleocytosis with lymphocytic predominance may also be observed, whereas the presence of more than 50 cells/mm^3^; the predominance of neutrophils, eosinophils or other atypical cells; and a high protein concentration should lead to alternative diagnoses (Table 1). Blood tests are typically normal, but can be used to exclude other diseases, such as systemic autoimmune disorders or infections.^17,39^ Recently, the kappa-free light chain (KFLC) index has been increasingly recognized as a valuable biomarker in the diagnosis of MS. Studies suggest that the KFLC index may be more sensitive and specific than traditional OCBs in identifying early MS, offering a faster, less subjective diagnostic alternative.^40–43^ Indeed, its proposed inclusion in the updated diagnostic criteria, as presented at the 2024 European Committee for Treatment and Research in Multiple Sclerosis (ECTRIMS) Congress,^32^ marks a significant step forward in the early diagnosis of MS. In addition, serum neurofilament light chain (NfL) and glial fibrillary acidic protein (GFAP) levels have emerged as potential biomarkers reflecting ongoing neuronal damage and disease activity, guiding treatment choices.^44,45^

#### Diagnostic criteria

The diagnosis of relapsing-remitting MS (RRMS) relies primarily on demonstrating dissemination in space (DIS) and DIT, as defined in the 2017 revised McDonald criteria. DIS is established by the presence of lesions in at least two of the four classical CNS regions: the periventricular, juxtacortical/cortical, infratentorial, and spinal cord regions. DIT can be demonstrated through clinical relapses or by MRI findings, such as the coexistence of enhancing and nonenhancing lesions or the appearance of new T2 lesions on follow-up scans. In patients with a single clinical event and evidence of DIS, CSF-specific OCBs may replace DIT. The diagnosis of primary progressive MS (PPMS) requires one year of clinical progression plus at least two of three supportive criteria (Table 2).^18^

**Table 2:** McDonald 2017 diagnostic criteria for multiple sclerosis

Category	Diagnostic criteria
**Dissemination in space (DIS)**	≥2 clinical attacks with objective evidence of ≥2 lesions OR MRI lesions in ≥2 characteristic CNS regions: - Periventricular - Cortical/Juxtacortical - Infratentorial - Spinal cord
**Dissemination in time (DIT)**	≥2 clinical attacks separated by ≥1 month OR MRI evidence of the simultaneous presence of gadolinium-enhancing and nonenhancing lesions OR New T2 lesions appearing on follow-up MRI OR Presence of CSF-restricted oligoclonal bands (in patients with clinical and radiological evidence of DIS)
**Diagnosis of RRMS**	Evidence of both DIS and DIT demonstrated clinically, via MRI, or with supportive CSF findings (oligoclonal bands)
**Diagnosis of PPMS**	≥1 year of disability progression independent of clinical relapses PLUS 2 of the following: -≥1 T2-hyperintense lesions characteristic of MS in periventricular, cortical/juxtacortical, or infratentorial regions -≥2 T2-hyperintense spinal cord lesions -Presence of CSF-restricted oligoclonal bands

*MRI* magnetic resonance imaging, *CNS* central nervous system, *CSF* cerebrospinal fluid, *RRMS* relapsing–remitting multiple sclerosis, *PPMS* primary progressive multiple sclerosis, *DIS* dissemination in space, *DIT* dissemination in time

In 2024, an international panel of experts proposed updated diagnostic criteria for MS, aiming to increase diagnostic accuracy and reduce the time to diagnosis. Although not yet formally published, these proposed 2024 McDonald criteria include several major changes: (1) the possibility of diagnosing radiologically isolated syndrome (RIS) as MS in specific high-risk scenarios; (2) formal inclusion of the optic nerve as a fifth CNS region for assessing DIS; (3) elimination of the requirement for DIT in selected cases; (4) acceptance of CSF KFLC as an alternative to OCBs to establish intrathecal inflammation and fulfill DIT; and (5) incorporation of advanced MRI biomarkers, such as CVS and PRLs, as supportive features to improve specificity. These refinements are especially relevant in atypical presentations and in special populations, such as patients over 50 years of age or those with headache or vascular comorbidities, where diagnostic uncertainty may be greater.

### Clinical phenotypes

MS phenotypes are traditionally classified on the basis of clinical progression and disease activity, with the most common form being RRMS, which is characterized by clearly defined relapses with full or partial recovery and stable periods between episodes. Approximately 50–80% of these patients develop secondary progressive MS (SPMS), which is characterized by gradual worsening of neurological function, within an average period of 15–20 years after disease onset.^28,46^ A smaller subset (15%) presents primary progressive MS (PPMS), which involves steady disease progression from onset. Clinically isolated syndrome (CIS) or first demyelinating syndrome is an initial clinical episode suggestive of demyelination but does not yet meet the full diagnostic criteria for MS. Additionally, a classification of active vs nonactive depending on the presence of relapses or radiological activity has been introduced for the progressive forms, thus facilitating treatment strategies since only active patients respond to MS therapies^28,47^; this paradigm could change owing to the recent publication of the HERCULES trial, which demonstrated the efficacy of tolerobrutinib in nonactive SPMS.^48^ Recently, the concepts of PIRA and relapse-associated worsening (RAW) have emerged, highlighting distinct mechanisms of disability accumulation. PIRA refers to disability progression that occurs independently of clinical relapses, emphasizing underlying neurodegenerative processes, whereas RAW represents disability worsening directly related to relapses. These distinctions are valuable both in clinical practice and in research, providing a better understanding of disease dynamics and tailoring therapeutic strategies.^49,50^

RIS refers to the incidental discovery of MRI findings suggestive of MS in individuals without clinical symptoms or signs consistent with demyelination. Individuals with RIS have an increased risk of developing CIS or definite MS over time. Factors predicting a greater risk of progression from RIS to symptomatic MS include spinal cord and infratentorial lesions and the presence of OCBs. The management of RISs remains an area of ongoing research, focusing primarily on monitoring and identifying high-risk individuals who might benefit from early intervention.^51^

Despite these classifications, some experts have encouraged neurologists to understand the disease as a continuum defined by the relative contributions of overlapping pathological and reparative/compensatory processes, which may vary across individuals and over time.^52^

Another clinically relevant phenotype, although defined by age of onset rather than disease course, is pediatric-onset multiple sclerosis (POMS). POMS is a rare but increasingly recognized chronic, immune-mediated demyelinating disorder of the CNS that presents before the age of 18. It accounts for approximately 3–5% of all MS cases and shares core pathological features with adult-onset MS (AOMS), including multifocal inflammation, demyelination, axonal loss, and neurodegeneration.^53,54^ However, POMS has distinct clinical and biological characteristics. Compared with adults, children typically experience a higher relapse rate, greater lesion burden on MRI, and more active gadolinium-enhancing lesions.^55^ Despite this aggressive presentation, neurological recovery tends to be more complete in early disease stages owing to heightened neuroplasticity in the developing CNS. Nonetheless, owing to their early onset, individuals with POMS often reach irreversible disability at a younger chronological age than those with AOMS.^54^

Notably, cognitive impairment, which affects domains such as processing speed, working memory, and executive function, is more prevalent in POMS patients. These deficits are attributed to both inflammatory demyelination and disrupted neurodevelopmental trajectories, particularly when disease onset occurs during critical periods of brain maturation.^56,57^ Such impairments can have long-lasting effects on academic achievement, psychosocial functioning, and quality of life, necessitating early cognitive screening and interdisciplinary support.

The diagnosis of POMS is based on the 2017 McDonald criteria, with adaptations validated for pediatric use. It requires evidence of DIT and DIS on MRI and is often supported by CSF findings such as the presence of OCBs. Importantly, alternative diagnoses that are relatively more frequent in younger children, such as acute disseminated encephalomyelitis (ADEM), neuromyelitis optica spectrum disorder (NMOSD), or genetic and metabolic leukoencephalopathies, must be carefully ruled out through clinical, radiologic, and serological assessment.^58–60^ Indeed, the proposed 2024 McDonald criteria strongly recommended myelin oligodendrocyte glycoprotein (MOG)-IgG testing via cell-based assays in children with a first CNS demyelinating event before the age of 12 and in those over 12 years with atypical presentations of MS to exclude MOG antibody-associated disease (MOGAD), which is among the most prevalent demyelinating conditions in pediatric populations.^32^ This recommendation is supported by evidence from multicenter studies,^61^ which reported that up to 39% of children with demyelinating syndromes were MOG-IgG positive.

The management of POMS involves both acute relapse treatment and long-term disease modification. High-dose intravenous corticosteroids remain the first-line therapy for acute exacerbations. Disease modifying treatments (DMTs) constitute the cornerstone of long-term management, with first-line agents including interferon (IFN)-β and glatiramer acetate. More recently, fingolimod became the first DMT approved specifically for pediatric MS in patients aged ≥10 years, following the PARADIGMS trial.^62^ In light of accumulating evidence, there is a growing consensus favoring early initiation of high-efficacy therapies in pediatric patients with high disease activity to reduce the inflammatory burden, preserve neurological function, and delay long-term disability.^63,64^

### Pathology and pathogenesis

The pathology of MS is characterized by multifocal areas of inflammation, demyelination and gliosis within the white and gray matter of the brain and spinal cord, known as plaques or lesions. The loss of myelin sheaths disrupts neuronal function and impairs nerve signal transmission.^65^ In the early inflammatory phases of the disease, such as CIS and RRMS, active demyelinating lesions predominate. These lesions exhibit lymphocyte infiltration, mainly of CD8^+^ T cells and B cells, with a lower presence of CD4^+^ T cells. Additionally, activated microglia and macrophages are observed at lesion edges, along with reactive astrocytes, contributing to ongoing inflammation and tissue damage.^66^ In contrast, in progressive forms, including PPMS and SPMS, inactive demyelinating lesions bestride. These lesions are well demarcated, with established demyelination, reduced axonal density, reactive astrocyte gliosis, and microglial activation, and unlike earlier stages, they display lower levels of lymphocyte infiltration.^67^ Despite reduced peripheral inflammation, chronic immune activation persists. Notably, tertiary lymphoid structures, consisting of plasma cells, B and T cells, and follicular dendritic cells (DCs), have been identified in the meninges, suggesting that sustained inflammation within the CNS plays a critical role in ongoing tissue damage and subsequently in disease progression.^68^

The etiology of MS remains elusive. In fact, many genetic and environmental factors are associated with MS development (see below). The pathogenesis of MS has also not been fully elucidated; however, both innate and adaptive immune responses are known to be involved. MS is driven primarily by autoreactive adaptive immune cells that infiltrate and promote damage within the CNS. Dysregulation of immune effector–suppressor cell interactions results in an autoimmune response against CNS antigens.^69^ The hypothesis about the autoimmune origin of MS derives from studies on experimental autoimmune encephalomyelitis (EAE), an experimental model of MS in which disease can be induced by immunization with CNS-derived proteins and is largely driven by CNS-specific CD4^+^ T cells.^70–76^ A critical event in MS immunopathogenesis is disruption of the BBB, which normally restricts immune cell entry into the CNS. Proinflammatory cytokines and matrix metalloproteinases degrade the BBB, allowing autoreactive lymphocytes to attack myelin sheaths, leading to demyelination and subsequent neuronal injury.^77–79^ Within the CNS, resident cells such as microglia and astrocytes also contribute to the inflammatory milieu. Activated microglia present antigens and secrete proinflammatory mediators, perpetuating the immune response.^80,81^ Astrocytes, which are traditionally considered supportive cells, have been shown to play roles in both promoting and resolving inflammation. Chronic activation of these cells can lead to sustained neuroinflammation, axonal damage, and the formation of sclerotic plaques characteristic of MS^82–85^ (see Immuno- and Neuropathogenesis of MS section).

## Etiology and hypotheses on MS pathogenesis

**Fig. 1:**
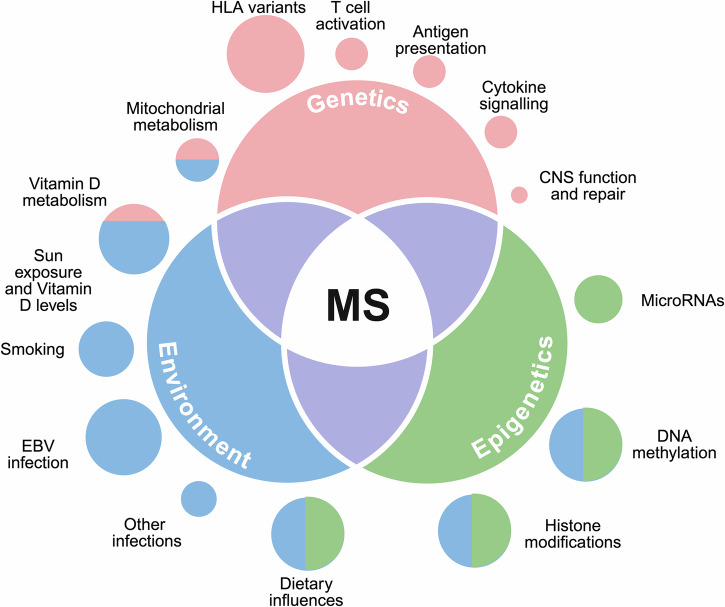
The interplay of genetic, environmental, and epigenetic factors in multiple sclerosis risk. Multiple sclerosis (MS) risk is influenced by the interaction of genetic susceptibility, environmental exposure, and epigenetic modifications. This Venn diagram illustrates how these three factors overlap, with MS emerging at their intersection. Genetics (pink) contributes to inherited susceptibility, with variations in immune-related genes playing a key role. Environmental factors (blue), such as infections, vitamin D levels, and smoking, can affect disease risk. Epigenetics (green) represents the dynamic regulatory mechanisms that mediate the effects of the environment on gene expression, shaping immune responses and disease progression. The surrounding bubbles represent the contributions of individual factors and their interplay, emphasizing the multifactorial nature of MS pathogenesis. Created in https://BioRender.com

While the specific etiology of MS remains elusive, its pathogenesis is clearly multifactorial and involves complex interactions between genetic predispositions and environmental influences (Fig. 1). Therefore, these contributing factors are typically categorized broadly into environmental triggers and genetic susceptibility, both of which have been shown to play significant roles in disease onset and progression.

In that sense, a recent meta-analysis using Canadian MS data developed a probabilistic model to infer the effect that the different factors have on the probability of developing MS, focusing on the likelihood that a randomly selected individual from the genetically susceptible population experiences an event that is, by itself, “sufficient” to trigger the disease. Interestingly, the results of this study suggest that MS development requires both an appropriate genetic background and a “sufficient” level of environmental exposure. However, even when both factors are present, MS may still not manifest.^15^ This observation suggests that, while both genetic and environmental factors are necessary for the development of MS, part of the genetic predisposition might stem from currently unknown stochastic immune processes, epigenetic reprogramming and/or somatic mutations.

### Genetic factors

Currently, the genetic component of MS risk is strongly supported by familiar and twin studies. For example, the lifetime risk of developing MS in the general population is approximately 0.1%, whereas in individuals with a first-degree relative affected by MS, it ranges between 2% and 4%.^86^ Additionally, the concordance rate among monozygotic twins is estimated to be between 20% and 30%, whereas dizygotic twins exhibit a lower concordance of approximately 5%. Parent‒child concordance is approximately 2%, which, while low, still represents a 10- to 20-fold increased risk compared with the general population.^87^

Over the past 20 years, extensive research (primarily through large genome-wide association studies [GWASs]) has greatly advanced our understanding of the genetic underpinnings of MS. These studies have highlighted the crucial role of genes involved in the adaptive immune system at MS onset, particularly those influencing T-cell function. The most well-established genetic association with MS involves the human leukocyte antigen (HLA) class II region, particularly the HLA-DRB1*1501 allele.^88^ This allele results in a significant increase in MS susceptibility and is implicated in antigen presentation and immune regulation. However, it is important to highlight that MS does not follow a simple Mendelian inheritance pattern, indicating the involvement of multiple genes with small effect sizes.^86^ In that direction, one of the most recent and largest MS susceptibility GWASs, which leveraged genotypes from 47,429 MS cases and 68,374 controls, resulted in the identification of 233 statistically independent associations with MS susceptibility that were significant genome-wide.^89^ Among these variants, 32 were located in the HLA gene region. The remaining 200 genetic associations were autosomal, and the first X chromosome linked gene variants outside the HLA gene region, either within or near genes that are expressed in various peripheral immune cells and in resident immune cells of the brain.

Until 2019, MS genetics focused predominantly on identifying susceptibility variants associated with the development of the disease. As a result, we now understand 48% of its inheritability.^89^ However, in 2023, a progression MS GWAS was conducted, representing a significant advancement in the field.^90^ This study successfully identified the first genome-wide significant gene variant associated with MS progression. A variant in the dysferlin-zinc finger protein 638 (DYSF-ZNF638) locus was linked to a decrease in the median time to require a walking aid and increased brainstem and cortical pathology in brain tissue. Interestingly, none of the previously identified MS susceptibility variants were associated with MS progression or severity.

Among the more than 230 genetic variants associated with MS risk identified over the years via GWAS, most appear to be related or directly linked to immune system function^91^. When segregating HLA-related loci from non-HLA loci, distinct functional patterns emerge. HLA variants, particularly those in the HLA-DRB1 region, are predominantly involved in antigen presentation and adaptive immune responses, shaping the interaction between autoreactive T cells and antigen-presenting cells (APCs).^92^ In contrast, non-HLA variants are enriched across all major immune cell types, influencing various immunological pathways.

Functionally, these non-HLA risk loci can be categorized on the basis of their roles in MS pathogenesis. Several variants are associated with T-cell activation, cytokine signaling, and antigen presentation, affecting genes such as interleukin (IL)-2RA, IL17R,^93^ IL4, IL6,^94^ IL12,^95^ IRF5, CD24, CD58, and EVI5.^96^ Others are linked to vitamin D metabolism, including polymorphisms in the vitamin D receptor (VDR)^97^ and CYP27B1,^98^ an enzyme essential for vitamin D activation, highlighting an intersection between genetic susceptibility and environmental influences. Some risk variants are also found in mitochondrial DNA,^99,100^ implicating energy metabolism and oxidative stress in MS pathology. Finally, as mentioned earlier, the recent discovery of genes associated with CNS function and repair, including DYSF-ZNF638,^90^ apolipoprotein E (ApoE)^101^ and dipeptidyl peptidase 6 (DPP6),^102^ may influence neuroprotection and synaptic plasticity, which appear to be critical for disease progression.

Notably, most of these genetic risk factors are enriched across all major immune cell types, but some of them exert opposing effects depending on the immune cell type in which they are expressed, highlighting the complexity of MS pathogenesis.^103^ This underscores the need for further studies, including single-cell characterization, to delineate the context-dependent roles of these loci. Furthermore, the epigenomic landscapes of different immune and CNS-resident cells in MS-susceptible individuals remain largely unexplored, representing a crucial gap in our understanding of how genetic risk translates into disease manifestation.

Notably, in most genetic screenings, samples were collected in Europe and the USA, and it is important to acknowledge that MS genetics differ among populations. While it is likely that the majority of genetic risk factors are shared, as has been observed between European/American populations and African Americans,^104–106^ we cannot exclude the possibility that there are unidentified gene variants associated with MS susceptibility and/or progression that may be subpopulation specific; therefore, wider studies are still needed.

In summary, genetic studies have revealed that the onset of MS is driven primarily by immunological mechanisms predominantly in the adaptive arm of the immune system, whereas disease progression appears to be influenced by processes in the CNS. Nevertheless, all these insights set the stage for further functional studies, aiming to uncover the underpinnings of MS disease, paving the way for novel prevention strategies and personalized treatment on the basis of the diversity of genetic profiles of patients.

### Environmental triggers

While genetic predisposition plays a significant role in MS susceptibility, the disease does not follow a Mendelian inheritance pattern, as evidenced by its incomplete concordance among monozygotic twins and the influence of multiple low-effect genetic variants. These findings indicate that environmental exposure is a critical contributor to MS pathogenesis. Over the years, several epidemiological studies have provided compelling evidence that various environmental factors interact with genetic susceptibility to influence disease risk. Large multinational efforts, such as the Environmental Risk Factors in MS Study (EnvIMS),^107^ have highlighted the importance of exposure during adolescence, with factors such as Epstein–Barr virus (EBV) infection,^108^ smoking,^109^ sunlight exposure,^110^ vitamin D levels and dietary influences^111^ emerging as key determinants of disease risk.

One of the oldest and most well-documented observations is the latitudinal gradient of MS incidence, with higher disease incidence in populations residing at greater distances from the equator, which led, over 50 years ago, to a link being drawn with variation in levels of ultraviolet radiation (UVR).^112^ This geographic distribution has prompted researchers to investigate vitamin D deficiency as a potential contributing factor, as individuals living at higher latitudes receive less UVR exposure, resulting in lower endogenous vitamin D production and possibly greater MS susceptibility.

Higher vitamin D levels, particularly before the age of 20, have been associated with a lower risk of developing MS later in life,^113^ with additional evidence from the EnvIMS study, where supplementation and sun exposure support its protective effect.^111,114^ Recently, high vitamin D levels have also been shown to correlate with decreased axonal damage, as assessed by CSF NfL levels in patients with MS.^115^

Genetic studies further support the role of vitamin D in MS. Polymorphisms near CYP27B1, a key enzyme in vitamin D metabolism, have been linked to an increased risk of the disease.^116,117^ Additionally, case‒control studies using Mendelian randomization approaches have shown that genetic variants regulating vitamin D levels significantly influence MS susceptibility.^118,119^

Vitamin D has also been linked to MS progression and neurodegeneration. The administration of 1,25-dihydroxyvitamin D3 in the EAE model has demonstrated evidence of slowing disability progression. Notably, progression was even prevented, and upon re-exposure following vitamin D withdrawal, the effects suggested a reversible block of disability, highlighting its potential therapeutic role in MS.^118,119^ In patients, higher baseline 25-dihydroxyvitamin D (25(OH)D) levels (≥50 nmol/L) have been associated with slower disease progression, as evidenced by lower Expanded Disability Status Scale (EDSS) scores over the following four years and reduced annual brain volume loss.^120^ Similarly, an independent correlation has been observed between vitamin D deficiency and greater disability in MS patients, as reflected by higher EDSS scores, where low 25(OH)D levels in one cohort accounted for an 11.5% increase in EDSS scores,^121^ further reinforcing the influence of vitamin D status on MS disability progression.

From a pathogenic perspective, vitamin D is a key modulator of immune function, and its deficiency has been implicated in immune dysregulation, facilitating MS development via a decrease in the differentiation of effector T and B cells, the promotion of regulatory subsets, the modulation of innate immune cells and a reduction in immune cell trafficking that acts at the BBB.^122^ Furthermore, vitamin D has been implicated in both myelination and remyelination. Experimental studies suggest that remyelination is promoted by the regulation of oligodendrocyte precursor cell (OPC) differentiation while also attenuating demyelination,^123,124^ enhancing microglial activation and thus facilitating the clearance of myelin debris and creating a more favorable environment for repair.^125^

Interestingly, more than 80% of known MS-associated genes are enriched in vitamin D response elements (VDREs) in their promoter regions and, consequently, may be regulated by vitamin D.^126–128^ Moreover, VDREs have also been recently found to have differential open chromatin and methylation statuses in the promoter regions of these VDREs.^129,130^

Infectious agents have also been proposed as environmental triggers of MS, with EBV emerging as one of the most studied candidates. EBV has been implicated in the development of MS, with recent research showing that nearly all individuals who developed MS were EBV positive at the time of onset. A longitudinal study involving 801 MS patients from over 10 million U.S. military personnel reported that all but one had contracted EBV before developing MS, providing strong evidence that EBV is a trigger for the disease.^131^

The mechanisms by which EBV influences MS, which have been recently extensively reviewed,^132,133^ involve complex interactions that are still debated. Various hypotheses exist, including molecular mimicry, where EBV proteins cross-react with myelin components,^134^ and the ability of viruses to rescue autoreactive B cells, which could lead to autoimmunity,^135^ direct infiltration of EBV into the CNS, potentially inducing inflammation and immune responses that exacerbate MS,^136^ are among the most accepted. However, critical questions remain about how latent EBV infection in B cells interacts with MS risk alleles^136^ and how EBV-modulated immune responses contribute to disease progression, highlighting the need for further research into EBV as both a trigger and a potential driver of MS.

Other infections, including human herpesvirus 6 (HHV-6), cytomegalovirus (CMV), and human endogenous retroviruses (HERVs), have been investigated for their potential roles in MS. HHV-6 is highly prevalent and can establish lifelong infection early in life. Meta-analyses and case‒control studies suggest an association between HHV-6 infection and increased MS risk.^137,138^ CMV, another herpesvirus, shows a more complex relationship with MS. While a meta-analysis revealed no overall difference in CMV seroprevalence between patients with MS and controls, regional variations suggest a protective effect in Europe but an increased risk in the Middle East. Additionally, previous studies have suggested that CMV seropositivity may be protective against MS onset, although findings remain inconclusive regarding interactions with HLA genes.^139^ HERVs, remnants of ancient retroviruses, have also been implicated in MS pathogenesis, with MS-associated retrovirus (MSRV) proteins and nucleic acids found at significantly higher levels in patients with MS.^140^ Although some evidence links HERV reactivation to MS progression through T-cell activation and microglial inflammation, the limited sample size and geographic scope of studies warrant cautious interpretation.^141^ Similarly, Chlamydia pneumoniae^142–144^ has also been investigated for its potential role in triggering autoimmunity in susceptible individuals, although a definitive causal relationship still remains unproven.^137^

Smoking has also been associated with an increased risk of MS, likely owing to its effects on immune activation and chronic inflammation. A substantial body of high-quality evidence consistently links active tobacco smoking to a greater risk of MS onset,^145^ with a dose-dependent relationship—greater exposure, such as increased cigarette pack years, correlates with greater risk.^146^ In contrast, the impact of passive smoking remains uncertain, as studies have produced conflicting results. While a meta-analysis revealed no significant effect,^146^ a large case‒control study suggested a dose‒dependent association, with long-term exposure tripling MS risk.^147^

Beyond onset risk, numerous studies have investigated the link between smoking and MS progression, although the findings are not entirely consistent. Evidence suggests that smoking accelerates the transition from RRMS to SPMS and worsens disability progression, as demonstrated in case‒control and cohort studies across different populations.^148^

Mechanistically, smoking may contribute to MS progression through oxidative stress, nitric oxide-induced axonal degeneration, increased BBB permeability, and chronic immune activation.^149,150^ Additionally, epigenetic interactions with HLA genes further support the role of smoking in MS susceptibility, with smokers carrying HLA-DRB1*15 and lacking HLA-A02 facing a 13-fold increased risk.^151^

In addition, nutritional factors have emerged as key modulators of MS risk and progression, with increasing evidence highlighting the impact of diet on immune regulation, neuroinflammation, and the gut microbiome composition.^152–155^

Obesity, particularly in early life, has been linked to increased MS risk and neuroinflammation, largely through adipokine-induced immune modulation and vitamin D deficiency.^156–159^ In RRMS, obesity is correlated with greater disability, elevated IL-6 and leptin, reduced IL-13 in CSF, and an altered lipid profile associated with worse outcomes.^160^ A higher body mass index (BMI) has also been linked to greater brain atrophy, a predictor of long-term disability.^161^ However, its role in MS progression remains unclear, with some studies suggesting a higher risk in obese males but not females and an increased likelihood of SPMS conversion at age 20—although only in smokers.^162–164^

In contrast, dietary interventions such as the ketogenic diet and caloric restriction have shown promise in experimental models and clinical trials by modulating immune responses, reducing oxidative stress, and promoting neuroprotection.^165–168^ The composition of fatty acids in the diet also plays a crucial role, with omega-3 polyunsaturated fatty acids exerting anti-inflammatory effects, whereas excessive intake of saturated fats may contribute to neuroinflammation.^169,170^

Additionally, the gut microbiome, which is shaped by dietary habits, has gained attention for its role in regulating immune tolerance and influencing MS susceptibility.^171–173^ A recent study revealed how MS patients exhibit gut microbiome heterogeneity, with enriched *Blautia* and *Akkermansia* species and a reduced *Bifidobacterium* to *Akkermansia* ratio, which is linked to disease severity. This ratio, validated in both mouse models and human cohorts, has emerged as a potential microbial marker for predicting MS severity and guiding microbiome-based interventions.^174^

Another recent meta-analysis highlighted a common gut microbiome alteration across autoimmune neurological diseases, including MS, characterized by a reduction in short-chain fatty acid (SCFA)-producing bacteria (such as *Faecalibacterium* and *Roseburia*) and an increase in pathogenic or opportunistic microbes (*Streptococcus*, Escherichia–Shigella).^172^ Given that SCFAs play a key role in histone acetylation and epigenetic regulation, their depletion may contribute to immune dysregulation and neuroinflammation. A ketogenic diet, known to increase SCFA levels, has been proposed to counteract these effects by restoring the histone acetylation balance and modulating immune responses, potentially offering therapeutic benefits in MS and related conditions.^175,176^ Notably, infiltrating CD4^+^ T cells in the CSF of MS patients cross-react with gut microbial peptides from MS-associated bacterial genera that have high homology with human peptides, suggesting a role for the gut microbiota in MS pathogenesis.^177^

### Hypotheses on MS etiology: inside-out vs. outside-in

While MS pathogenesis remains a topic of debate, two predominant paradigms, the “outside-in” and “inside-out” hypotheses, have shaped the discussion of MS origins for years.^178^ The outside-in model proposes that an aberrant autoimmune response initiated in the periphery leads to CNS damage, whereas the inside-out model posits that a primary neurodegenerative process within the CNS triggers a secondary immune response against myelin debris. As MS is a syndrome with multiple clinical presentations rather than a single disease entity, it is likely that both immune-mediated (outside-in) and neurodegenerative (inside-out) mechanisms contribute to disease initiation in MS patients with different phenotypes (see Immuno- and Neuropathogenesis in MS section). Importantly, these mechanisms are not mutually exclusive; both immune and neurodegenerative processes play integral roles, regardless of the initiating event, differing primarily in their temporal sequence.^179^

From a genetic point of view, the results indicate that the majority of MS risk loci are associated with immune system function,^180^ whereas only a few have been linked to oligodendrocyte maturation or neuronal degeneration,^181^ supporting the notion that dysregulated immune responses, rather than a primary CNS-mediated process, are influenced by genetic predispositions and environmental triggers, and play a dominant role in MS onset and progression. As detailed in the following section, several molecular pathways involved in the immune pathogenesis of the disease further emphasize the pivotal involvement of immune-mediated mechanisms in disease onset and progression.

From a phenotypic perspective, while RRMS appears to be largely driven by peripheral immune responses targeting the CNS, progressive forms of MS seem to be mediated by intrinsic CNS immune processes behind an intact BBB,^67,182,183^ characterized by SELs, chronic microglial activation, and cortical demyelination, with little to no signs of meningeal inflammation.^67^ In contrast, in SPMS, tertiary lymphoid structures resembling lymphoid follicles containing B and T lymphocytes, macrophages, and plasma cells are frequently observed in the meninges.^184^ Additionally, progressive MS is also associated with increased spinal cord lesion burden and gray matter atrophy, which are correlated with disability severity.^183,185,186^ Evidence from well-validated nonhuman primate models of MS suggests an endogenous trigger for the disease, aligning with the inside-out model. These models demonstrate that myelin sheath dissociation (blistering) in normally appearing white matter leads to the systemic release of modified (citrullinated) myelin antigens, potentially triggering autoimmunity. For example, T-cell hyperreactivity to citrullinated MOG has been shown both in these models and in MS patients.^187^

In parallel, other studies have suggested two distinct neuropathological findings as early indicators of MS: “microglial nodules”, which involve damaged axons surrounded by small clusters of macrophages/microglia,^188^ and “newly forming lesions”, characterized by apoptotic oligodendrocytes.^66^ Neither of these features initially include T-cell infiltration or overt demyelination,^189^ further supporting the neurodegenerative (inside-out) hypothesis.

Nonetheless, rather than viewing the outside-in and inside-out models as opposing theories, an integrated perspective that accommodates both mechanisms may provide a more comprehensive framework for understanding MS etiology and pathogenesis. This approach acknowledges the actual interplay between immune-mediated and neurodegenerative pathogenic processes, as thoroughly discussed in the following section, and underscores the necessity of considering both pathways when developing therapeutic strategies.

## Immuno- and neuropathogenesis in MS

### Immune tolerance

While activation of the immune system is essential for defending the body against pathogens, combating cancer, and managing inflammatory conditions, excessive or dysregulated immune activity can lead to detrimental outcomes such as autoimmune disorders, allergies, and hypersensitivity reactions. To prevent such adverse effects, the immune system must maintain a finely tuned balance between mounting effective defenses against external insults and avoiding harmful responses to self-antigens. This critical equilibrium is achieved through immune tolerance, a fundamental system that prevents the immune system from attacking self-antigens while allowing robust responses against pathogens. Immune tolerance occurs through both central and peripheral mechanisms that regulate immune homeostasis and prevent autoimmunity.^190^

The capacity of the immune system to recognize a wide range of foreign peptides relies on the high diversity of immune cell receptors. While this diversity is crucial for preventing infections, it also inherently increases the risk of generating receptors that target self-peptides, potentially leading to autoimmune diseases. To mitigate this risk, the immune system employs central and peripheral selection mechanisms as critical checkpoints for maintaining immune tolerance and preventing autoreactivity.^191^ Central tolerance occurs during T and B-cell development in the thymus and bone marrow, respectively. This process aims to eliminate or neutralize self-reactive lymphocytes, thereby preventing autoimmunity. In the thymus, T-cell progenitors undergo rigorous selection on the basis of the affinity of their T-cell receptors (TCRs) for the self-peptide-(p)-major histocompatibility complex (MHC). Double-positive (CD4^+^CD8^+^) thymocytes in the cortex, whose TCRs bind to pMHCs, are positively selected and subsequently migrate to the medulla. Within the medulla, T cells bearing high-affinity TCRs for self-antigens are eliminated through clonal deletion, while those with intermediate affinity differentiate into regulatory T (Treg) cells. This selection process is facilitated by specialized thymic cells, including cortical and medullary thymic epithelial cells (cTECs and mTECs), DCs, and B cells, which present a diverse array of self-antigens. These presentations are guided by transcription factors such as autoimmune regulator (AIRE) and forebrain embryonic zinc finger-like protein 2 (FEZF2).^192^

Within the bone marrow, developing B cells undergo selection on the basis of B-cell receptor (BCR) affinity for self-antigens. High-affinity binding triggers clonal deletion, whereas lower-affinity binding can induce anergy, a state of functional unresponsiveness. A significant portion of B cells edit their BCRs to avoid self-reactivity through ongoing immunoglobulin light (L) chain gene recombination, which alters antigen specificity. B cells that fail to edit or correct heavy (H) chain features may undergo apoptosis. Therefore, central tolerance regulates autoreactive B cells, reducing their frequency, affinity for self-tissue, or functionality within the B-cell repertoire.

Despite the mechanisms of central tolerance, some self-reactive lymphocytes are found in the periphery in nondisease conditions, necessitating additional peripheral tolerance mechanisms to maintain immune homeostasis,^191,192^ which are crucial in preventing immune reactions to self-antigens and hypersensitivity reactions to innocuous antigens encountered outside primary lymphoid organs. Key processes in peripheral tolerance include activation-induced cell death (AICD), anergy and suppression by Treg cells. Since T-cell activation requires three distinct signals: TCR recognition by pMHCs, costimulation (e.g., the CD28-CD80/86 interaction), and cytokine signaling, the absence of costimulation or the presence of inhibitory signals during antigen recognition can induce AICD or T-cell anergy.^192,193^ AICD in CD4^+^ T cells is triggered by repeated stimulation, a process accompanied by high levels of IL-2 production. This leads to the coexpression of Fas (CD95) and Fas ligand (FasL), and subsequent Fas-FasL engagement initiates T-apoptosis.^194^ Conversely, anergy in T cells is induced by the interaction of coinhibitory receptors, such as programmed cell death protein (PD)-1 and cytotoxic T-lymphocyte antigen (CTLA)-4, with costimulatory molecules; prolonged antigen exposure; and the production of anti-inflammatory cytokines, such as IL-10, transforming growth factor (TGF)-β, and IL-35.^195–198^ Similarly, B cells require both BCR engagement and secondary signals for full activation; high-avidity BCR interactions without appropriate costimulation can lead to B-cell anergy or deletion.^199^ The precise mechanism governing the divergence of self-reactive T and B cells after TCR or BCR activation in the absence of costimulation remains unclear.^191^

In addition, Treg cells play a central role in peripheral tolerance, with major subtypes including FoxP3^+^ cells and IL-10-producing (Tr1) cells.^200^ Treg cells differentiate in the thymus (tTregs) in response to self-antigens and migrate to peripheral tissues to limit autoreactivity and promote tissue repair. Additionally, they can also differentiate from naive CD4^+^ T cells in the periphery (pTregs) to enforce tolerance to antigens not expressed in the thymus, such as food, allergens, microbial, or pregnancy-associated antigens. Furthermore, tissue-resident Treg cells in various tissues exhibit specialized phenotypes and functions.^191,192,200^ Tr1 cells, another important Treg subset, are IL-10^+^FoxP3^−^CD4^+^ T cells. Their differentiation is induced by factors such as IL-27 and modulated by host and microbial metabolites. Tr1 cells suppress autoreactivity via different mechanisms that halt T-cell and APC responses, including the production of IL-10 to control the general activation of immune responses; the secretion of perforin and granzyme B to eliminate myeloid APCs; the expression of the inhibitory coreceptors CTLA4 and PD1, which inhibit the proliferation of autoreactive T cells; and mechanisms mediated by the expression of the ectoenzimes CD39 and CD73.^201^

Finally, DCs are pivotal in establishing both central and peripheral immune tolerance. By processing and presenting antigens, DCs orchestrate T-cell differentiation and dictate T-cell commitment to a specific pathway, inducing anergy or deletion through the provision of diverse cytokines and stimulatory or inhibitory molecules.^202^ DCs contribute to immune regulation through multiple mechanisms, including the downregulation of costimulatory molecules (CD80, CD86, and CD40), the expression of inhibitory molecules (PD-L1, inducible T-cell co-stimulator ligand [ICOSL], and B and T lymphocyte attenuator [BTLA]), the suppression of proinflammatory cytokine production (IL-6, IL-12, IL-23, and tumor necrosis factor [TNF]), and the production of anti-inflammatory cytokines (IL-10, TGF-β, and IL-27) and immunomodulatory metabolites (IDO, retinoic acid, and lactate).^200,203^

Taken together, central and peripheral immune tolerance involve the participation of numerous cell types. The interplay between these mechanisms ensures effective immune responses against pathogens while maintaining tolerance to self-antigens and innocuous environmental antigens. However, under conditions such as MS, this equilibrium is disrupted. MS highlights the paradox of immune tolerance breakdown within the CNS despite its immune privilege.^204–207^

#### Immune privilege of the CNS

CNS immune privilege is generally understood as a mechanism to protect nonregenerative neural tissues from damage caused by complex immune responses. This concept originated from observations that tissue grafts in the brain are not rejected^208,209^ and is supported by distinct anatomical features of the CNS, including the BBB, which restricts immune cell infiltration through tight junctions, efflux transporters, and low levels of leukocyte adhesion molecules; the absence of classical lymphatic drainage, which limits antigen surveillance and immune activation in draining lymph nodes; and the limited antigen-presenting capacity of microglia and the relative scarcity of professional APCs in the brain parenchyma.^204–207,210^

The concept of CNS immune privilege has evolved significantly in recent years, revealing a more nuanced understanding of the interactions between the CNS and the immune system. This new perspective highlights the dynamic equilibrium between neuroprotection and immune engagement rather than absolute isolation. The CNS maintains antigenic surveillance through a sophisticated system involving CSF drainage and immune activity in the dura mater.^211^ CNS-derived antigens are continuously flushed into the CSF and mixed with interstitial fluid to create a dynamic reservoir of neural waste.^211^ This fluid then accesses the dura mater through perivascular pathways along cerebral veins, where it accumulates near the dural sinuses.

Advanced imaging studies, including fluorescent tracers in rodents and Gd-based MRI in humans, have confirmed that CNS antigens are captured by dural APCs, including DCs, macrophages and B cells. These APCs then prime circulating T cells, enabling peripheral immune recognition of CNS perturbations.^211–213^ This mechanism is particularly evident in EAE, where myelin-specific T cells activated in the dura migrate to the leptomeninges and parenchyma, driving neuroinflammation.^214^

This new understanding of CNS immune interactions is further supported by recent research on the meningeal lymphatic system. The discovery of functional meningeal lymphatics revealed their role not only in fluid homeostasis but also in facilitating immune cell migration from the CNS to the periphery.^215^ These lymphatic vessels provide a pathway for immune cells, particularly DCs, to carry CNS antigens to cervical lymph nodes, coordinating immunity through antigen delivery.^210,216^ Complementing this drainage system, the dura mater itself serves as a crucial interface for immune surveillance and B-cell development in the CNS. Recent research has revealed that the dura mater contains not only mature B cells but also developing B cells, including pro, pre, and immature B cells, a function previously thought to be exclusive to the bone marrow.^217,218^ This lymphopoietic niche is supported by stromal cells around the dural sinuses that produce developmental ligands such as CXCL12 and IL-7.^216,217^ Constant exposure to CNS antigens in the meninges facilitates the elimination of autoreactive B cells, particularly those recognizing CNS-specific antigens such as myelin MOG.^210,217^ This process provides an additional site for immune tolerance that is distinct from that of the peripheral bone marrow. Furthermore, the dura mater harbors IgA-producing plasma cells, which are educated in the gut before migrating to the meninges.^219^ These cells play a critical role in protecting the brain from blood-derived pathogens, highlighting the importance of local immune responses at the borders of the brain in preventing CNS infections.^219^

Recent research has revealed a novel mechanism of neuroimmune communication centered on a repertoire of CNS-derived regulatory self-peptides presented on MHC-II molecules.^220^ These guardian self-peptides, which are found along the brain’s lymphatic drainage pathways during homeostasis, play crucial roles in maintaining immune tolerance. When their presentation is enhanced, they expand a population of suppressor CD4^+^ T cells that control CNS autoimmunity through CTLA-4- and TGF-β-dependent mechanisms. Notably, during neuroinflammatory conditions, the presentation of these regulatory self-peptides diminishes, potentially contributing to disease progression.^220^

The concept of CNS immune privilege is evolving from a model of passive isolation to one of active immunoregulation. Recent studies have shown that neuroimmune interactions occur at CNS borders, such as meningeal tissues and cervical lymph nodes, rather than through complete segregation. These interfaces employ specialized mechanisms to balance immune surveillance with neuronal protection, reflecting the unique needs of neural tissues. This shift emphasizes that CNS immune privilege arises from biological adaptations rather than from inaccessibility, redefining our understanding of neuroinflammatory diseases.^210^

#### Tolerance breakdown mechanisms

Despite the intricate mechanisms governing immune tolerance, the system remains imperfect, allowing for the emergence of autoimmune disorders. Consequently, extensive research has focused on uncovering the breakdown mechanisms driving neuroimmune dysregulation. Recent advances in neuroimmunology have revealed several key pathways that potentially contribute to the disruption of the delicate balance between the nervous and immune systems. These emerging hypotheses not only shed light on the complex interplay of factors involved in neuroinflammatory conditions but also offer promising avenues for therapeutic interventions. Among the emerging mechanistic hypotheses of immune tolerance breakdown, genetic and epigenetic factors play crucial roles, with variants in genes such as HLA-DRB1 altering microglial antigen presentation and immune tolerance pathways.^221^ Molecular mimicry between pathogen antigens and CNS proteins may trigger autoimmune responses. The structural similarity between EBV nuclear antigen 1 (EBNA1) and the CNS ion channel Anoctamin 2 (ANO2) could promote neuroinflammation in conditions such as MS.^222,223^ Additionally, checkpoint dysregulation, such as dysfunction of inhibitory receptors such as LAG-3 in meningeal lymphatics, may fail to suppress autoreactive T cells, exacerbating CNS autoimmunity.^224^ Furthermore, an imbalance between Treg and T helper (Th)17 cells disrupts immune equilibrium at the BBB, whereas mitochondrial stress in microglia leads to metabolic reprogramming that sustains proinflammatory states.^225^ These interconnected mechanisms highlight the complexity of neuroimmune interactions and their role in disease pathogenesis, offering new opportunities for targeted therapeutic approaches.

### Immunopathogenesis

Despite our incomplete understanding of the etiology and pathogenesis of MS, classifying this disease as an autoimmune disorder is widely accepted. As already mentioned, the autoimmune origin of MS is based on evidence obtained from the EAE model, with which it shares clinical, pathological, and histological similarities.^74,226–230^ EAE is a CD4^+^ T-cell-mediated condition that is experimentally induced by immunizing animals with CNS-derived antigens, leading to immune-mediated demyelination and subsequent motor dysfunction characterized by ascendent paralysis that affects the hind limbs and forelimbs.^227,228^ On the basis of EAE studies, the most accepted hypothesis for MS immunopathogenesis suggests that peripheral autoreactive CD4^+^ T cells become activated and migrate into the CNS, where they are reactivated by CNS antigens, triggering an immune attack toward myelin, destroying the myelin sheath and ultimately leading to axonal loss.^231,232^ In support of this hypothesis, studies using transgenic humanized mice expressing MS-associated HLA haplotypes and myelin-specific TCRs derived from human CD4^+^ T cells demonstrated the relevance of CD4^+^ T cells. These mice not only develop EAE symptoms after active immunization but also exhibit spontaneous neurological dysfunction.^233–235^ For example, it has been widely demonstrated that not all CD4^+^ T cells that recognize self-antigens are deleted in the central tolerance process that takes place in the thymus; therefore, autoreactive CD4^+^ T cells are present in the circulation in patients with MS, as well as in healthy controls. Nonetheless, the repertoire of myelin autoreactive CD4^+^ T cells from MS patients differs from that of healthy controls, presenting higher antigen avidity and showing a skewed proinflammatory profile that was not observed in samples from healthy controls.^236,237^

Regardless of the evident role of CD4^+^ T cells in MS pathogenesis, the mechanisms underlying their activation in the periphery remain a subject of debate. One of the hypotheses suggests that autoreactive CD4^+^ T cells might be triggered in the periphery by CNS-derived antigens. One possibility relies on the capacity of APCs that are present in the brain parenchyma and that have direct access to CNS antigens to leave the CNS and present CNS antigens in the periphery. Alternatively, soluble CNS antigens carried in the CSF could be transported to the nasal mucosa and then drain to cervical lymph nodes, where they would be presented by APCs to autoreactive CD4^+^ T cells, which in turn would be activated. A second hypothesis states that autoreactive CD4^+^ T cells are activated in the periphery by molecular mimicry, meaning cross-recognition of microbial and/or viral peptides or gut microbial antigens with high homology with CNS/myelin epitopes.^177,238–240^ A recent investigation reported that the population of autoreactive peripheral CD4^+^ T cells exhibits a high prevalence of brain-homing T cells capable of recognizing an antigen expressed both in B cells and the CNS, potentially elucidating a mechanism by which T cells are initially activated in the periphery and subsequently reactivated against the same antigen within the CNS.^241^ A third hypothesis points to a failure in the peripheral regulatory mechanisms of tolerance in MS patients. In this context, Treg cells are the main immune cell type involved in the maintenance of peripheral tolerance. Although the frequency of Tregs in MS patients is similar to that in healthy controls, their suppressor function against autoreactive CD4^+^ T-cell responses is impaired.^242,243^ The resistance of effector T cells to Treg cell suppression has also been described as a mechanism of MS pathogenesis. For example, effector T cells from RRMS patients with active disease are resistant to suppression by FoxP3-Treg cells, which is mediated by an increase in IL-6 signaling and downstream activation of signal transducer and activator of transcription (STAT)3.^244^ In parallel, a subset of self-antigen-specific Treg cells lost the expression of FoxP3 in the context of autoimmunity activation in the EAE model, supporting the notion that, in MS, the immunoregulatory function of Treg cells is impaired and fails to control the autoimmune process.^245^

**Fig. 2:**
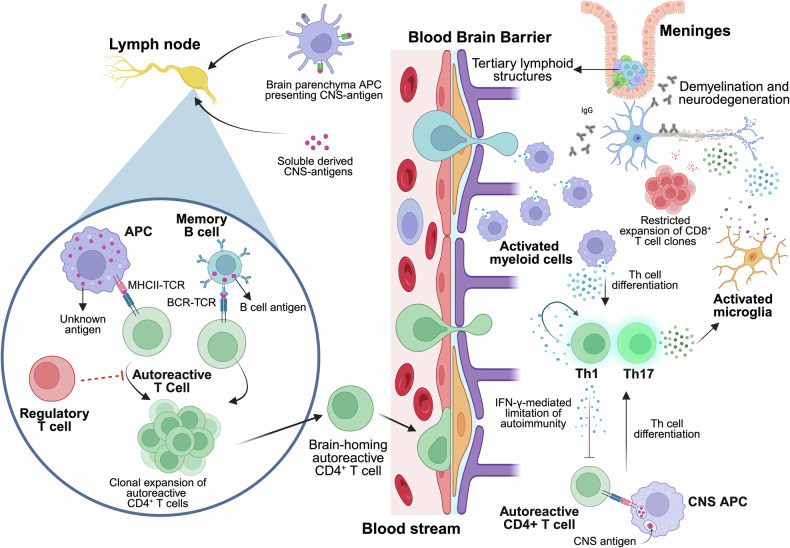
Immunopathogenesis of multiple sclerosis. Although the antigen responsible for the initiation of the autoimmune reaction in multiple sclerosis (MS) has not yet been described, it is hypothesized that antigens derived from the central nervous system (CNS) would reach the periphery and be presented to autoreactive CD4^+^ T cells in the lymph nodes. Alternatively, other self-antigens, pathogenic or gut microbial peptides sharing high homology with myelin antigens, have been postulated as candidates for autoimmune MS. The pool of autoreactive CD4^+^ T cells, which are enriched in brain-homing clones, circulate inside the CNS upon activation. Peripheral tolerance mechanisms involving the suppressor functionality of regulatory T (Treg) cells, including reduced suppressor function of Treg cells, loss of FoxP3 or resistance of effector T cells to Treg-mediated suppressive mechanisms, have also been shown to be dysregulated in MS patients. Once inside the CNS, autoreactive CD4^+^ T cells are reactivated by local antigen-presenting cells and differentiate into encephalitogenic T helper (Th)1/Th17 effector cells, initiating a series of immunological events that lead to demyelination and neuronal damage. Myeloid cells are the most abundant immune cell type found in MS lesions and are involved in multiple aspects of MS pathogenesis, including the differentiation of CD4^+^ T cells and the recruitment of other immune cells at the lesion site. CD8^+^ T cells are also recruited into the CNS and are clonally expanded, although the heterogeneity of clones is much greater than that of CD4^+^ T-cell clones. The formation of tertiary lymphoid structures similar to germinal centers in the meninges results in the activation of T and B cells, along with the sustained secretion of antibodies. APC, antigen-presenting cell; BCR, B-cell receptor; CNS, central nervous system; IFN- γ, interferon gamma; MHC-II, class II major histocompatibility complex; IgG, immunoglobulin isotype G; TCR, T-cell receptor; Th, T helper cell. Created in https://BioRender.com

A variety of Th autoreactive CD4^+^ T cells with different cytokine release patterns are involved in MS pathogenesis. Th1 cells produce IFN-γ but also other proinflammatory mediators, such as IL-2 (to sustain the survival of T cells) and TNF-α. Although IFN-γ is primarily thought to be pathogenic in the context of MS, the deletion of the cytokine or the receptor in transgenic knockout (KO) mice exacerbates EAE clinical symptoms and results in the accumulation of autoreactive CD4^+^ T cells in the CNS, demonstrating that IFN-γ is necessary to limit autoimmunity by inhibiting T-cell proliferation and inducing the apoptosis of activated CD4^+^ T cells.^246^ Some years later, Th17 cells were identified as a novel Th subtype of pathogenic cells whose differentiation and expansion are dependent on IL-23. Th17 CD4^+^ T cells present a proinflammatory secretion pattern different from that of Th1-IFN-γ-producing cells, characterized by the production of IL-17A, IL-17F, granulocyte‒macrophage colony-stimulating factor (GM‒CSF), IL-6, IL-21, IL-22, TNF-γ and the cytolytic enzyme granzyme B.^247,248^ Importantly, Th17 cells were identified as essential players in the establishment of CNS inflammation in EAE.^248^ Subsequent studies revealed the expression of IL-17 in the brains of MS patients, specifically in perivascular lymphocytes and glial cells. Strikingly, active lesions contain a greater abundance of IL-17-producing CD4^+^ and CD8^+^ T cells than inactive MS lesions do.^249^ IL-17 was also shown to promote BBB disruption and facilitate the migration of Th17 cells into the CNS, where they directly contribute to tissue damage^247^; reviewed in ref.^250^ These emerging data highlight the relevance of IL-17 and Th17 cells in MS, completely changing the established paradigm of Th1-driven MS immunopathogenesis (Fig. 2).

While CD4^+^ T cells are pivotal in MS onset and progression, CD8^+^ T cells also play a significant role, as they are abundant in the inflammatory infiltrate of MS lesions. Notably, the density of CD8^+^ T cells was positively correlated with the extent of axonal damage. Importantly, unlike CD4^+^ T cells, CD8^+^ T cells infiltrating MS lesions exhibit a much more homogeneous clonal composition and are restricted to a few expanded clones.^251^

A hallmark of MS diagnosis is the presence of IgG OCBs in the CSF; however, the specificity of these antibodies remains largely unknown. Although antibodies against myelin components, specifically MOG and myelin basic protein (MBP), are detectable in the CSF and serum of MS patients, their presence in the CSF is not exclusive to MS. Although anti-MOG antibodies were initially associated with conversion to MS, they were later linked to a distinct entity, MOGAD, rather than MS.^252,253^ Nevertheless, the formation of tertiary lymphoid structures in the meninges of MS patients, where B and T cells interact and B cells mature, indicates sustained secretion of antibodies into the CNS and strongly supports the contribution of B cells to the immunopathogenic mechanisms of MS.^254^

B-cell-depleting therapies with anti-CD20 antibodies are very efficient at suppressing disease activity in MS patients; however, antibody-secreting plasma cells do not express CD20, suggesting that B cells may contribute to MS immunopathogenesis through other functions beyond the production of pathogenic antibodies. For example, B cells serve as APCs and have been identified as key players in the activation of proinflammatory T cells (Th1 and Th17) in the context of EAE.^255^ Studies with peripheral blood mononuclear cells (PBMCs) from MS patients demonstrated that CD4^+^ T cells exhibit increased autoproliferation, a process that is mediated by memory B cells presenting self-antigens through the MS-associated HLA-DR15 haplotype and the secretion of proinflammatory cytokines.^241,256,257^ Regarding the production of proinflammatory cytokines, a study identified a subset of circulating proinflammatory memory B cells that produce GM-CSF along with TNF-α and IL-6 and drive proinflammatory myeloid cell activation through GM-CSF. The authors reported that these GM-CSF-producing B cells are present at a relatively high frequency in untreated MS patients. Moreover, GM-CSF production was associated with STAT5/6 signaling, which was found to be increased in the B cells of untreated MS patients. Notably, B-cell depletion therapies restored the activation of the signaling pathway to normal levels and restored the frequency of GM-CSF-producing B cells and the subsequent development of IL-10-regulatory B cells, suggesting a new mechanism to explain the success of anti-CD20 therapies in MS.^258^ Together, these findings underscore the complex role of B cells in MS and strongly suggest that their pathogenic potential extends beyond antibody production, involving antigen presentation and local immune regulation within the CNS.

Myeloid cells, including microglia, monocytes, macrophages and dendritic cells, play a central role in MS pathogenesis. They contribute to neuroinflammation through antigen presentation to CD4^+^ T cells, phagocytosis and the release of proinflammatory cytokines, chemokines that recruit lymphocytes, and reactive oxygen species (ROS) that contribute to tissue injury and mitochondrial dysfunction (reviewed in ref. ^259^). Inflammatory infiltrates in active MS lesions are composed primarily of myeloid and T cells, with cells of the myeloid lineage representing the most prevalent population.^260,261^ Within these lesions, myeloid cells promote lymphocyte infiltration and contribute to neurodegeneration through the production of cytotoxic mediators and the induction of oxidative stress.^262^ Additionally, local microglia process and present CNS antigens through MHC class II and contribute to the reactivation of infiltrating autoreactive CD4^+^ T cells^263^; however, microglia can exhibit both pro- and anti-inflammatory phenotypes^264^ and contribute to the clearance of myelin debris,^259^ thereby contributing to both acute lesion formation and remyelination.^265^ In early active MS lesions, approximately 40% of phagocytic cells are proinflammatory microglia,^266^ and together with CD8^+^ T cells, these cells mediate myelin destruction, axonal injury, and BBB disruption.^67^ Chronic microglial activation persists in mixed active/inactive lesions (also referred to as smoldering lesions), which are associated with neurodegeneration.^267^ In progressive MS, microglia may drive neurodegeneration through cytokine release, glutamate excitotoxicity, and oxidative damage.^184^

Myeloid cells localized near active MS lesions in brain tissues activate the STAT3 signaling pathway, which is involved in multiple cellular processes, including T-cell differentiation. Furthermore, STAT3 signaling in myeloid cells is essential for promoting the differentiation of myelin-specific pathogenic Th1/Th17 cells in the early stages of EAE through antigen processing and presentation and the production of inflammatory cytokines,^268^ suggesting a principal role of myeloid cells not only in the propagation of the pathogenic autoimmune response inside the CNS and the neurodegenerative process but also in the establishment of the disease in the periphery.

### Neuropathogenesis

**Fig. 3:**
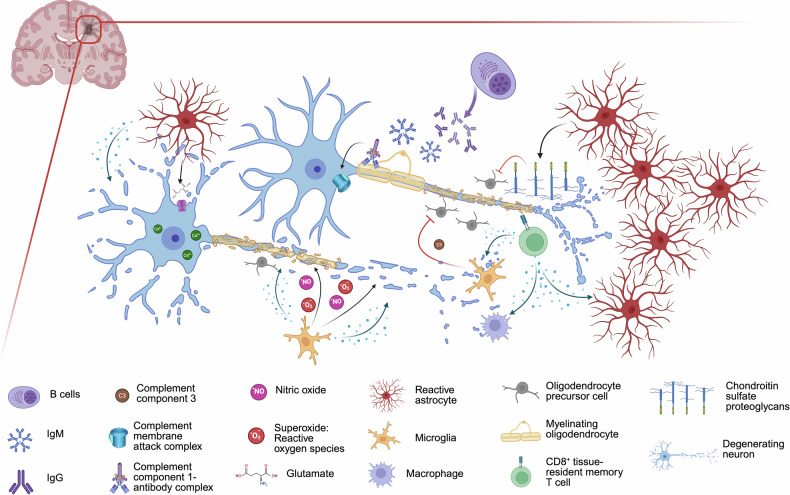
Neuropathogenesis of multiple sclerosis. Multiple sclerosis (MS) is caused by demyelination, which begins with microglial activation, macrophage infiltration, and accumulation of CD8^+^ tissue-resident memory T cells, which drive inflammatory changes in astrocytes and microglia. Chronic microglial activation leads to the release of proinflammatory cytokines and reactive oxygen and nitrogen species, which contribute to demyelination, tissue damage and neurodegeneration. Similarly, chronically reactive astrocytes release cytokines and chemokines that recruit additional immune cells, deposit inhibitory chondroitin sulfate proteoglycans that impair remyelination, and contribute to glial scar formation, which further hinders repair. Humoral immunity also plays a role in demyelination through antibody deposition and complement activation. IgG and IgM antibodies trigger the classical complement pathway, leading to opsonization, formation of the membrane attack complex (MAC), and direct damage to oligodendrocytes and neurons. In addition, the C3 component impairs remyelination. The failure of oligodendrocyte progenitor cells to mature into fully functional oligodendrocytes further exacerbates remyelination deficits. Over time, chronically demyelinated neurons experience axonal injury and metabolic stress, causing calcium accumulation within axons, activation of proteases, and consequently axonal degeneration. Additionally, chronic inflammation exacerbates neurodegeneration by exposing neurons to cytotoxic molecules such as nitric oxide and glutamate excitotoxicity. Together, these mechanisms drive progressive tissue damage and disease progression in MS. Created in https://BioRender.com

MS is characterized by the loss of myelin sheaths surrounding axons, which leads to a broad range of neurological impairments contributing to the wide array of symptoms observed in MS patients, including motor and sensory deficits, cognitive impairment, and fatigue.^269^ However, the underlying neuropathogenesis of MS is much more complex and involves a combination of immune system dysregulation, neurodegeneration, and glial cell activation (Fig. 3).

Demyelination is one of the hallmark features of MS, and it refers to the loss or damage of the myelin sheath. In MS, demyelination occurs in the CNS as a result of immune-mediated attacks on oligodendrocytes, leading to the formation of plaques and lesions that disrupt nerve conduction.^270^

During the acute phase of MS, demyelination and plaque formation are accompanied by microglial activation and infiltration of macrophages and lymphocytes.^271,272^ However, in approximately 20% of lesions, inflammation persists and becomes more organized, marked by the accumulation of tissue-resident CD8^+^ memory T cells and monocytes. These cells promote inflammatory changes in astrocytes and microglia, leading to chronic tissue damage and remodeling.^273,274^

This chronic inflammation is especially prominent in mixed active and inactive lesions, a term that combines previously described chronic active, smoldering, and SELs.

Many of these lesions can be identified on high-field MR images because of the accumulation of iron-laden phagocytes at the lesion edge, bordering normal-appearing white matter.^275^

Humoral immunity has been shown to contribute to demyelination in MS through antibody deposition and complement activation. IgG and IgM antibodies, likely produced by clonally expanded B cells in the CNS, target myelin components, although no single dominant autoantigen has been identified.^276–279^ These antibodies can directly induce demyelination and activate the classical complement pathway, leading to inflammation, opsonization, and formation of the membrane attack complex (MAC), which damages oligodendrocytes.^280^ While complement-driven myelin destruction is well documented, some components, such as C3, may also impair remyelination, highlighting the complex balance between the pathogenic and potentially protective roles of complement in MS.^280^

While early-disease acute lesions may experience some degree of remyelination, this process is often incomplete in long-standing plaques. From a cellular perspective, acute active lesions exhibit BBB breakdown, perivascular infiltration of primarily cytotoxic T cells, widespread presence of microglia and macrophages, and varying degrees of axonal damage. In contrast, chronic active lesions feature a gliotic, hypocellular core surrounded by a rim of activated myeloid cells. Last, chronic inactive lesions, on the other hand, have low cellularity throughout and are characterized by the accumulation of chondroitin sulfate proteoglycans.^281^

Recent findings indicate that a major barrier to remyelination in MS is the failure of OPCs to mature into fully functional oligodendrocytes. As shown in animal models, effective remyelination requires the proliferation, migration, and maturation of OPCs into myelinating oligodendrocytes. For example, in inactive and mixed active-inactive MS lesions, OPCs are present but in reduced numbers and are unevenly distributed, whereas mature oligodendrocytes are almost entirely absent,^282,283^ suggesting that impaired oligodendrocyte differentiation plays a critical role in remyelination failure in chronic lesions.^284,285^

Several factors contribute to this impairment, including chronic inflammation, the presence of inhibitory molecules within lesions, and aging, and further research indicates that both OPCs and mature oligodendrocytes may contribute to lesion repair, but the mechanisms underlying remyelination failure are complex and influenced by disease duration, lesion stage, and location.^286–289^

Over time, chronically demyelinated neurons are vulnerable to axonal injury and metabolic stress, contributing to neurodegeneration and disease progression.^290,291^ From a mechanistic point of view, the loss of myelin sheaths also induces an ionic imbalance in axons due to the resulting anomalous distribution of ion channels along the axons after demyelination. Consequently, calcium accumulates inside the axon, stimulating axonal proteases and leading to catalytic axonal proteolysis.^292–294^ This ionic imbalance has been recently detected through a few MRI studies in MS patients, which revealed an elevated sodium concentration in acute and chronic lesions compared with areas of nonlesioned white matter.^295,296^

Studies have shown that neuroaxonal injury occurs in both relapsing–remitting and progressive forms of MS, suggesting that neurodegeneration may be an intrinsic part of the disease process, not simply a consequence of accumulated demyelination.

One of the most striking findings is that neurodegeneration in MS is not confined to the later stages of the disease but can begin early in the course of MS, even before overt clinical symptoms appear, but becomes more obvious in tissue samples from patients with progressive disease.^297,298^ Owing to axonal damage, NfL is released into the interstitial space and eventually reaches the CSF and bloodstream. Its levels are correlated with disease activity, including relapse and progression, making it a valuable biomarker. NfL is now commonly used in clinical trials and is increasingly being integrated into routine practice to assess MS progression and treatment response.^44^

The mechanisms underlying neurodegeneration in MS are still being investigated, but several factors have been identified. Chronic inflammation is thought to play a role, as immune cells that infiltrate the CNS can directly damage neurons through the release of cytotoxic molecules such as nitric oxide,^299^ ROS,^300^ and glutamate excitotoxicity, the process by which excessive glutamate leads to neuronal injury.^301^ Furthermore, there is evidence that mitochondrial dysfunction and altered axonal transport also contribute to neurodegeneration in MS.^302,303^

As previously mentioned, microglia are the first cells to respond to injury or inflammation in the CNS. In MS, microglial activation is a hallmark feature of both acute and chronic lesions. While microglia are important for clearing cellular debris and modulating the immune response, their chronic activation in MS leads to the release of proinflammatory cytokines, ROS, and other neurotoxic factors that contribute to tissue damage and neurodegeneration.^281^ In that sense, the activation of microglia has been shown to exacerbate neurodegeneration in MS, is often observed in the vicinity of demyelinated lesions and may contribute to the ongoing injury of neurons and axons.^304^

Interestingly, recent studies have suggested that microglial activation is more pronounced in progressive forms of MS, where it is thought to play a key role in the ongoing neurodegeneration observed in these patients.^281^

In addition, astrocytes, which are the most abundant glial cells in the CNS, become activated in response to inflammatory signals and injury in MS. Activated astrocytes contribute to neuroinflammation by releasing cytokines and chemokines that recruit additional immune cells.^305,306^ However, they also play dual roles in protecting neurons and promoting tissue repair. Astrocytes can produce neurotrophic factors that support neuronal survival and remyelination, but chronic activation of astrocytes can lead to the formation of glial scars, which inhibit the repair process and contribute to tissue damage.^273,305,307^

The neuropathogenesis of MS involves a complex interplay between immune cell activation, demyelination, neurodegeneration, and glial cell responses.^302^ Understanding the mechanisms underlying neurodegeneration and the dual nature of glial activation is crucial for developing more effective treatments. As research continues to evolve, a more nuanced understanding of MS pathogenesis will hopefully lead to therapies that can not only halt disease progression but also promote tissue repair and functional recovery in affected individuals.

## Treatment strategies

### Acute treatment

Although the primary goal in the management of MS is to prevent relapses, understanding the indications and therapeutic options for acute treatment is essential. Acute therapy accelerates clinical recovery but does not appear to reduce long-term disability or prevent future relapses. Consequently, treatment decisions must consider relapse severity, patient clinical history, comorbidities, and previous responses to acute treatments. General recommendations include treatment of moderate-to-severe relapses in the absence of contraindications, whereas mild relapses without significant functional impairment may not require treatment.^29,308^ First-line acute treatment consists of high-dose systemic corticosteroids, typically methylprednisolone at doses of 500–1000 mg per day, which are administered intravenously or orally, as both administration routes have shown comparable clinical efficacy and safety profiles.^309,310^ The treatment duration generally ranges from 3–5 days, although it may be extended by another 3–5 days if significant clinical recovery is not achieved initially. Compared with MS, plasma exchange should be reserved for severe relapses refractory to corticosteroid treatment, which is more common in other demyelinating conditions, such as NMOSD or MOGAD.^31^

### Approved disease-modifying therapies

**Fig. 4:**
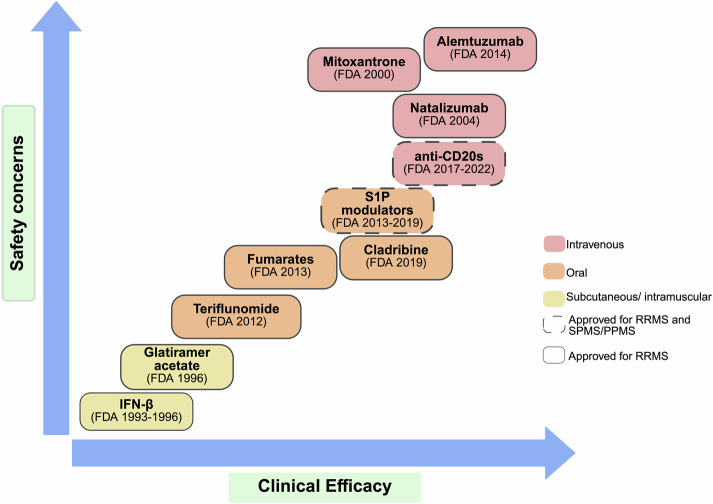
Disease-modifying therapies (DMTs) approved for multiple sclerosis. The figure represents the different DMTs according to their clinical efficacy (horizontal axis) and safety concerns (vertical axis). The colors indicate the route of administration: subcutaneous or intramuscular (yellow), oral (orange), or intravenous (red). The dashed borders denote DMTs approved for both relapsing-remitting multiple sclerosis (RRMS) and either secondary progressive MS (SPMS) or primary progressive MS (PPMS). FDA Food and Drug Administration. Created in https://BioRender.com

DMTs encompass numerous treatments aimed at reducing relapse frequency and preventing short-term disability accumulation, thus improving the quality of life of MS patients. Before the Food and Drug Administration (FDA) approval of the first DMT [IFN-β-1b] in 1993, MS treatment relied on broad-spectrum immunosuppressive drugs such as azathioprine, methotrexate, mycophenolate mofetil, intravenous immunoglobulin, and corticosteroids. However, these treatments have limited clinical efficacy and low safety profiles, prompting the development of more targeted therapies. Advances in our understanding of MS pathophysiology have led to the approval of numerous DMTs, enabling a more personalized therapeutic approach. DMTs vary widely in their mechanisms of action, clinical efficacy, safety profiles, and administration routes. Most of them modulate or suppress immune activity to reduce relapse frequency and MRI lesion accumulation, but no therapy has definitively halted or significantly slowed neurodegeneration. Consequently, most DMTs are approved for RRMS, while options are limited for PPMS patients, with ocrelizumab showing modest efficacy.^311^ The following sections categorize DMTs by administration route, which generally correlates with efficacy and safety profiles (injectable DMTs have lower efficacy and better safety, whereas intravenous DMTs have higher efficacy but increased risks of adverse events). Figure 4 illustrates the approved MS therapies according to their clinical efficacy, safety profile, and administration routes.

#### Injectable DMTs

Injectable DMTs are administered subcutaneously or intramuscularly and include the interferon family and the amino acid copolymer glatiramer acetate. As mentioned earlier, IFN-β-1b was the first DMT approved for MS. Subsequently, IFN-β-1a and different IFN-β-1b formulations were introduced, offering both subcutaneous and intramuscular administration routes.^312^ The mechanisms of action of these drugs are not completely understood but likely involve immunomodulation through downregulating the expression of MHC molecules on APCs, decreasing proinflammatory cytokines and increasing anti-inflammatory cytokines, inhibiting T-cell proliferation, and blocking the trafficking of inflammatory cells to the CNS.^313^ Glatiramer acetate, a synthetic polypeptide administered subcutaneously, mimics MBP and shifts the immune response toward an anti-inflammatory profile.^314^ Both interferons and glatiramer acetate have modest clinical efficacy, reducing relapse rates by approximately 30% compared with placebo. Common adverse effects include injection site reactions, flu-like symptoms with interferon, and immediate postinjection reactions with dyspnea, chest tightness, palpitations and anxiety with glatiramer administration.^312,315^

#### Oral DMTs

Oral DMTs encompass drugs taken orally but with different mechanisms of action, clinical effectiveness and adverse effects: teriflunomide, fumarates, sphingosine-1-phosphate (S1P) receptor modulators and cladribine.

##### Teriflunomide

Teriflunomide is the active metabolite of leflunomide, a classic immunosuppressant in rheumatologic diseases, and is administered once daily. Teriflunomide inhibits dihydroorotate dehydrogenase, an enzyme involved in pyrimidine synthesis, thus reducing the proliferation of activated T and B lymphocytes. Compared with placebo, clinical trials have shown comparable effectiveness to injectable DMTs, with a 30% reduction in the annualized relapse rate. Its main advantage is oral administration, but some serious adverse events, such as hepatotoxicity and teratogenesis, should be considered with caution. Common adverse effects include headache, increased liver enzymes, diarrhea, nausea and alopecia. It is contraindicated during pregnancy and requires a washout procedure with cholestyramine if needed^316,317^).

##### Fumarates

This family includes two compounds: dimethyl fumarate and diroximate fumarate. These fumaric acid derivatives exert their immunomodulatory effects by activating the nuclear factor erythroid 2-related factor 2 (Nrf2) pathway, which regulates cellular responses to oxidative stress and inflammation. They have demonstrated efficacy in reducing relapse rates by 44% and 53%, respectively, in clinical trials.^318^ The posology is two doses per day, and common adverse effects include flushing, gastrointestinal symptoms (more frequent with dimethyl than with diroximel), and a risk of lymphopenia, necessitating periodic blood monitoring.^318,319^ Anecdotic cases of progressive multifocal leukoencephalopathy (PML), a rare but potentially fatal opportunistic infection caused by the JC virus, have been reported, with risk closely associated with the degree of lymphopenia. This highlights the importance of close monitoring in these patients.^320^

##### S1P modulators

These drugs function by modulating S1P receptors, which are involved in lymphocyte trafficking. By preventing lymphocyte egress from the lymph nodes, they reduce the infiltration of autoreactive immune cells into the CNS, thereby decreasing inflammation and disease activity in MS.^321^

Fingolimod was the first S1P modulator approved for MS after it was demonstrated to significantly reduce the relapse rate by 54% and MRI activity. It is administered once daily and is well tolerated, although it requires first-dose cardiac monitoring owing to the risks of bradycardia and atrioventricular block. Other potential adverse effects include macular edema, liver enzyme elevation, and increased risk of infection (including exceptional cases of PML).^322^ Siponimod demonstrated rates of relapse reduction similar to those of fingolimod and, importantly, delayed disability progression, making it the first therapy approved for active SPMS. Importantly, the cardiac effects are diminished compared with those of fingolimod, and first-dose monitoring is recommended only for patients with heart conditions.^323^ Ozanimod was subsequently approved for RRMS and was demonstrated to have the best tolerability and safety profile but apparently lower efficacy than their analogs.^324^ More recently, ponesimod, a selective S1P1 modulator, was approved as the latest drug in this class for relapsing forms of MS. In the OPTIMUM trial, ponesimod showed superiority over teriflunomide in reducing the annualized relapse rate and MRI lesion activity, while also demonstrating a significant benefit on fatigue outcomes. The main safety considerations are similar to those of the other S1P modulators, including transient bradycardia at treatment initiation, elevated liver enzymes, risk of infections, and rare cases of macular edema.^325^

##### Cladribine

Cladribine is a purine analog that selectively depletes B and T lymphocytes, leading to prolonged immunomodulation.^326^ It is administered orally in short treatment cycles (two courses over two years) and is indicated for RRMS patients with moderate–high activity. Clinical trials demonstrated a significant reduction in relapse rates of 58%, MRI lesion burden, and disability progression. Cladribine does not require continuous treatment, which can be advantageous for adherence. However, it is associated with an increased risk of lymphopenia, infections, and potential malignancies, necessitating regular blood monitoring and cancer screening before initiation.^327^

#### Intravenous DMTs

Since the approval of natalizumab in 2004, followed by alemtuzumab in 2014 and ocrelizumab shortly thereafter, monoclonal antibodies have represented a groundbreaking advance in MS treatment. Compared with previous treatments, these therapies have demonstrated a significantly greater reduction in relapse rates, both compared with placebo and active comparators. However, they also present a more challenging safety profile, complicating individualized treatment decisions. Although not classified as a monoclonal antibody, mitoxantrone is an intravenously administered DMT and therefore will also be discussed in this section.

##### Natalizumab

Natalizumab is a monoclonal antibody that inhibits immune cell migration into the CNS by blocking the α4-integrin subunit, an adhesion molecule expressed on the surface of lymphocytes that is involved in transmigration across the endothelium into the CNS.^328^ It has demonstrated high efficacy in reducing relapse rates by 68% and MRI lesion activity, particularly in patients with highly active RRMS. However, its use is associated with an increased risk of PML. The risk of PML is linked to prolonged natalizumab exposure, prior immunosuppressant use, and JC virus seropositivity. Regular monitoring with JC virus antibody testing and MRI surveillance is recommended to mitigate this risk.^329^ The standard interval between doses is 4 weeks, although extension to 6 weeks seems to reduce the risk of PML without significantly compromising the clinical effectiveness.^330^ Another potential serious complication is “rebound” disease activity after treatment discontinuation, thus requiring, in most cases, bridge therapy when necessary.^331^ Recently, a subcutaneous formulation with similar clinical effects and safety profiles has been approved, facilitating the adherence to treatment and comfort of patients.^332^

##### Anti-CD20 therapy

Ocrelizumab is a humanized monoclonal antibody that targets the CD20 antigen on B cells, leading to B-cell depletion and modulation of the immune response. It is infused once every 6 months. Ocrelizumab was the first therapy approved for PPMS based on the ORATORIO trial, which demonstrated a reduction in disability progression.^333^ Compared with IFN-β-1a, it is also highly effective in RRMS, reducing relapse rates by 47% and MRI lesion activity.^334^ Recently, another humanized anti-CD20 drug (ofatumumab), administered monthly via subcutaneous injection, demonstrated a similar clinical profile, providing an alternative with a more convenient dosing regimen.^335^ More recently, ublituximab, a glycoengineered chimeric anti-CD20 monoclonal antibody with enhanced antibody-dependent cellular cytotoxicity, has been approved for relapsing forms of MS. It is given as a short intravenous infusion every six months, with phase III trials showing significant reductions in relapse rates and MRI activity compared to teriflunomide^336^. Ocrelizumab, ublituximab, and ofatumumab require monitoring for infusion-related reactions, risk of infection, and screening for hepatitis B reactivation. Long-term safety data indicate a potential association with hypogammaglobulinemia, necessitating regular immunoglobulin level assessments.^333,334^ Rituximab, another anti-CD20 monoclonal antibody, appears to be clinically similar to the other anti-CD20 drugs^337^). However, no official agency has approved its use as a DMT for MS patients thus far.

##### Alemtuzumab

Alemtuzumab is a highly potent monoclonal antibody that targets CD52, a glycoprotein expressed on the surface of mature lymphocytes, with the consequent depletion of both T and B lymphocytes through antibody-dependent cellular cytotoxicity and complement-mediated lysis. It is approved for highly active relapsing MS and is typically reserved for patients with an inadequate response to other DMTs. It is administered in two courses 12 months apart. Compared with IFN-β-1a, clinical trials have demonstrated significant reductions in relapse rates and MRI lesion burden, with some evidence of sustained efficacy beyond initial treatment cycles.^338^ However, alemtuzumab carries substantial risks, including secondary autoimmune diseases (e.g., thyroid disorders, immune thrombocytopenia, nephropathies), infusion-related reactions, and increased susceptibility to infections. Recently, several postmarking cases of cerebrovascular complications have been reported. Long-term safety monitoring, including regular blood and urine tests, is required for at least four years posttreatment.^339^

##### Mitoxantrone

Mitoxantrone is a cytotoxic anthracenedione that inhibits topoisomerase II, leading to DNA damage and the suppression of T and B lymphocytes. It was approved in 2000 for aggressive forms of MS with activity. However, its use is limited by serious adverse effects, including dose-dependent cardiotoxicity and an increased risk of secondary acute myeloid leukemia. Owing to these risks, its cumulative lifetime dose is restricted to 140 mg/m², and patients require regular cardiac monitoring before and during treatment.^340,341^

The management of MS follows a personalized approach based on disease activity, severity, and individual patient factors. The main treatment strategies include the following:

- Early and high-efficacy treatment strategy: This approach advocates initiating treatment with high-efficacy DMTs (e.g., monoclonal antibodies, cladribine) early in the disease course to prevent relapses, limit disability accumulation, and improve long-term outcomes. This strategy is particularly favored for patients with aggressive MS phenotypes.^342^
- Escalation therapy: This traditional approach starts with lower-efficacy, first-line therapies (e.g., interferons, glatiramer acetate, or oral DMTs) and escalates to more potent treatments (e.g., monoclonal antibodies or immunosuppressants) if disease activity persists or worsens.
- Induction therapy: A strategy where patients are treated initially with a highly potent DMT (e.g., alemtuzumab) to induce long-term disease suppression, followed by lower-intensity maintenance therapy if needed.

Some important factors, such as drug pharmacodynamics, biological effects, the risk of adverse effects, “rebound” activity or patient preferences (e.g., pregnancy planning), should be considered before starting or switching therapies.

Regarding treatment duration, a recent randomized clinical trial concluded that discontinuing medium-efficacy therapy might be a reasonable option for patients older than 55 years with nonactive MS.^343^ A more recent observational study revealed a greater risk of disease activity in patients older than 50 years with nonactive MS after the discontinuation of fingolimod or natalizumab, similar to the “rebound” effect reported in younger patients. In contrast, they did not find a greater risk of relapse in the anti-CD20 therapy discontinuation group, suggesting the possibility of stopping treatment in these patients.^344^

### Treatment of symptoms

While DMTs are designed to target the underlying inflammatory process of MS, symptomatic treatment also plays a crucial role in improving quality of life and managing long-term disability. Symptomatic therapy is tailored to the specific needs of each patient and often requires a multidisciplinary approach. The key areas of symptomatic treatment include the following:

- Fatigue: Fatigue is one of the most common and disabling symptoms of MS and can be a direct consequence of the disease or secondary to comorbid factors such as depression or sleep disorders. Treatment strategies include lifestyle modifications, energy conservation techniques, and physical therapy. Classical pharmacologic options such as amantadine, modafinil, and methylphenidate have not been demonstrated to be superior to placebo in a recent randomized clinical trial.^345^
- Spasticity and muscle stiffness: Muscle stiffness and spasticity can significantly impair mobility and produce pain. First-line treatments include baclofen, tizanidine, and gabapentinoids. In refractory cases, botulinum toxin injections or intrathecal baclofen pumps may be considered.^346^
- Neuropathic pain and sensory symptoms: Neuropathic pain, including burning or electric shock-like sensations, is commonly treated with gabapentin, pregabalin, or duloxetine. Carbamazepine may be used for trigeminal neuralgia.^347^
- Bladder and bowel dysfunction: Overactive bladder symptoms are managed with anticholinergic medications (e.g., oxybutynin, solifenacin) or beta-3 agonists (mirabegron). Intermittent self-catheterization may be required for urinary retention. Constipation is treated with dietary modifications, fiber supplements, and laxatives.^348^
- Cognitive impairment and mood disorders: Cognitive dysfunction may benefit from cognitive rehabilitation programs. Depression and anxiety, which are common in MS patients, are managed with selective serotonin reuptake inhibitors (SSRIs) or cognitive behavioral therapy (CBT).^349^
- Gait and mobility impairment: Fampridine, a potassium channel blocker, can improve walking speed in some MS patients. Regular physical therapy and assistive devices also play essential roles in maintaining mobility.^350^
- Tremor and coordination problems: Tremor in MS can be challenging to treat, but options include propranolol, primidone, or, in severe cases, deep brain stimulation.^351^

A multidisciplinary approach involving neurologists, physiotherapists, occupational therapists, urologists, and mental health professionals is essential for optimal symptom management in MS patients.

### Current phase II and III clinical trials

The emergence of numerous targeted therapies has revolutionized the prognosis of patients with MS in recent years. However, some issues remain challenging, especially the limited options for progressive forms of the disease. For this reason, ongoing research continues to explore novel DMTs for MS, particularly focusing on new mechanisms of action that can increase treatment efficacy while minimizing adverse effects. One of the most promising classes under investigation is Bruton’s tyrosine kinase inhibitors (BTKis), which target B-cell and myeloid cell activation, offering potential benefits in both relapsing and progressive forms of MS.^352^

#### Bruton’s tyrosine kinase inhibitors

BTKi's work by modulating B-cell receptor signaling and myeloid cell activation, potentially reducing both acute inflammation and smoldering neurodegeneration. Several BTKi candidates are currently in phase II and III trials:

- Evobrutinib: Demonstrated a reduction in MRI activity in relapsing MS (RESCUE trial) but failed to confirm a reduction in the relapse rate of RRMS compared with teriflunomide in two multicentric trials (evolutionRMS1 and evolutionRMS2). Moreover, the incidence of serious adverse events (especially liver-related complications) was greater in patients receiving evobrutinib.^353^
- Tolebrutinib: In two phase III clinical trials (GEMINI 1 and GEMINI 2; ClinicalTrials.gov numbers NCT04410978 and NCT04410991, respectively), tolebrutinib did not demonstrate superiority over teriflunomide in reducing the annualized relapse rate in patients with RRMS.^354^ However, in a separate phase III placebo-controlled trial evaluating its efficacy in both relapsing and progressive forms of MS, tolebrutinib demonstrated a significant delay in disability progression in patients with nonrelapsing SPMS. Specifically, the HERCULES trial (ClinicalTrials.gov number NCT04411641) reported a 31% reduction in the risk of 6-month confirmed disability progression compared with placebo. Additionally, 8.6% of patients treated with tolebrutinib (nearly twice as many as those in the placebo group) experienced confirmed disability improvement. These findings highlight the potential of tolebrutinib as a groundbreaking therapy for nonactive SPMS, a condition with limited treatment options.^48^
- Fenebrutinib: In the phase II FENopta trial, fenebrutinib significantly reduced new gadolinium-enhancing T1 lesions and new/enlarging T2 lesions over 12 weeks compared with placebo. In the subsequent open-label extension, patients maintained very low annualized relapse rates (≈0.04–0.06), with more than 95% remaining relapse-free at up to 96 weeks. No disability progression was observed, and MRI activity remained minimal. The safety profile was consistent with prior data, with no new safety signals reported. Phase III trials (FENhance 1 and 2 for relapsing MS; FENtrepid for PPMS) are ongoing^355^.
- Other BTKi candidates, such as remibrutinib and orelabrutinib, are in phase II or III trials, but no results have been reported yet.

#### Other emerging therapies

- Remyelination therapies: Agents such as opicinumab (anti-LINGO-1) aim to promote myelin repair, although early trials have yielded mixed results.^356^
- Neuroprotective agents: The phosphodiesterase-4 inhibitor Ibudilast has shown potential in slowing brain atrophy in progressive MS, as demonstrated in the SPRINT-MS trial.^357^
- Next-generation monoclonal antibodies: Current efforts are directed toward developing more selective and safer B-cell and T-cell targeted therapies, with the goal of enhancing efficacy while reducing immunosuppressive risks.

These investigational therapies could significantly expand the MS treatment landscape, particularly for progressive forms, where current options remain limited. Continuous monitoring of trial outcomes will determine their future clinical application.

### Hematopoietic stem cell transplantation

Hematopoietic stem cell transplantation (HSCT) has been used for the treatment of MS and other autoimmune disorders since the late 1990s.^358–362^ It is based on the transplantation of healthy hematopoietic stem cells (HSCs) obtained from bone marrow, peripheral blood or cord blood to substitute malfunctioning or damaged immune cells of a given patient. The rationale for the use of HSCT in patients with MS is that the treatment depletes both adaptive and innate immune cells associated with the disease, followed by de novo reconstitution of the immune system, which allows the restoration of immune tolerance and leads to long-term suppression of inflammatory activity.^363^ There are two types of HSCT: allogenic HSCT and autologous HSCT (aHSCT). Both types of HSCT differ in the source of HSCs: allogeneic HSCs come from a matched donor (related or not related), whereas autologous HSCs involve the use of HSCs from the same patient. Allogeneic HSCT is not indicated for MS patients since the risk of adverse events is much greater than that of HSCT: a greater probability of graft-versus-host disease (GVHD) is associated with an attack of the grafted immune cells toward healthy cells from the patient, a greater risk of infection due to persistent immunosuppression to avoid GVHD, and a higher probability of graft failure. For these reasons, aHSCT is the procedure of choice for the treatment of autoimmunity, especially MS.^364^ HSCs are generally obtained via leukapheresis, and patients are previously treated with a combination of chemotherapy and granulocyte colony-stimulating factor (G-CSF) to eliminate T cells from the graft and improve HSC yields.^365^ aHSCT also involves the use of preconditioning treatments with chemotherapeutics to efficiently eliminate immune system cells, allow transplanted HSCs to be properly grafted in the host and reset the immune system. For the treatment of MS, high-intensity protocols are not recommended because of their high toxicity profile,^366^ but evidence of the efficacy of low-intensity conditioning procedures is lacking. Currently, the most commonly used treatments for MS are intermediate-intensity conditioning regimens, with bis-chloroethylnitrosourea, carmustine (BCNU), etoposide, ara-C, and melphalan (BEAM)-antithymocyte globulin (ATG) or cyclophosphamide-ATG regimens being recommended in the current European Society of Blood and Marrow Transplantation (EBMT) guidelines because of their better balance between risk and benefit. Notably, the process itself is associated with adverse effects, including the development of secondary autoimmunity, increased risk of infections and reactivation of viruses (EBV, cytomegalovirus).^367^

The first aHSCT for treating MS included patients with severe disability, RR and progressive forms of MS and/or rapidly evolving disease. The results of this first study pointed to a promising therapeutic application of aHSCT; although the follow-up periods were short (ranging from 3–36 months), the number of patients was limited (6–26 included patients), and patients presented severe disability. For example, Fassas et al. reported EDSS improvement in 6 out of 15 patients, although 3 experienced relapses or worsening disability.^358^ In 2001, Mancardi and colleagues reported the results of a clinical trial including 10 patients with rapidly evolving SPMS and described clinical stabilization but no clinical improvement after HSCT.^361^ The clinical trial by Burt and collaborators described the absence of new Gd-enhancing lesions after HSCT and neurological improvement in all patients. Although none of the patients presented changes in the EDSS score, the authors reported improvements of more than 10 points on the Scripps neurologic rating scale (NRS)^368^ in those patients who had lower EDSS scores and presented Gd-enhancing (active) lesions before HSCT.^360^ Despite the inclusion of high-risk MS patients, this study highlighted the importance of treating patients with lower EDSS scores and evidence of inflammatory activity prior to aHSCT. In 2003, Nash and collaborators published the results of a multicenter pilot study that included 26 highly disabled MS patients (EDSS range 5.0–8.0), the majority of whom had progressive forms of the disease (25 out of 26) and who underwent a high-dose immunosuppressive conditioning regimen followed by HSCT between 1998 and 2001. Disease stabilization was observed in 14 out of 25 progressive (SP and PP) patients. Importantly, the EDSS score of the only RRMS patient included in the study decreased by 0.5 points with respect to baseline.^362^

Retrospective multicenter studies have collected evidence of the efficacy of aHSCT in treating patients with MS.^369,370^ The principal limitation of retrospective studies relies on the heterogeneity of patients recruited to undergo HSCT and the high degree of disability at the time of treatment in trials conducted at the end of the 1990s and beginning of the 2000s, contributing to underscoring the efficacy of HSCT in treating patients with MS. Nevertheless, retrospective studies have identified risk factors associated with treatment failure. An observational, retrospective, multicenter study comprising 281 patients from 25 centers in 13 different countries that received HSCT from 1995–2006 reported that although the majority of patients included in this study presented with progressive MS, disease progression-free survival was observed in approximately 50% of patients 5 years post-HSCT, reaching 73% in the subgroup of RRMS patients and dropping to 33% in patients with SPMS. Importantly, the study highlights the importance of the number of DMTs received before aHSCT, given that the probability of progression is greater in those patients who were previously treated with more than three immunosuppressive or immunomodulatory treatments. Age and higher EDSS scores were also identified as factors associated with disease progression after aHSCT.^370^ Similarly, Boffa et al. reported evidence for the use of aHSCT in patients with aggressive MS despite being on treatment. The collected data presented the heterogeneity of patients (e.g., variable age, disease duration and EDSS score) and procedures. The observational, retrospective, multicenter cohort study included 210 patients with MS (including RR, SP and PPMS) treated in 20 different Italian centers from 1997–2019; these patients underwent up to seven different preconditioning treatments depending on the individual experience of each center, were followed up for a distinct period of time after aHSCT, and heterogeneous study endpoints were assessed. However, the study revealed that the beneficial effect of aHSCT has persisted for a decade. The EDSS score significantly decreased after 10 years of follow-up in RRMS patients, whereas the EDSS score stabilized but did not decrease in progressive MS patients. Moreover, the authors reported disability worsening-free survival in 65.5% of treated MS patients after 10 years of aHSCT, reaching 71% in RRMS, and concluded that aHSCT performs better in RR than in progressive MS. They also reported that the therapy is more effective in those patients who present a higher relapse rate (disease activity) the year before transplantation.^369^ In fact, the observation that aHSCT is more beneficial for RRMS than for SPMS has been confirmed in other studies.^371^

Clinical trials conducted in the middle 2000s refined the selection of patients and homogenized the end point assessments, as reported in real-world experience and retrospective studies.^372,373^ In the HALT-MS clinical trial, high-dose immunosuppression followed by aHSCT was assessed in a prospective, open-label, single-arm phase II clinical trial. In accordance with previous experience, this study recruited RRMS patients with lower EDSS scores (range 3.0–5.5) who did not respond to DMTs. The rates of event-free survival, defined as no clinical or radiological relapse and no disability progression, at 3 and 5 years post-HSCT were 78% and 69%, respectively.^374,375^ A single-center, prospective phase II clinical trial included both RRMS and SPMS patients who did not respond to conventional DMTs. The 3-year event-free survival rate after aHSCT was 60% in the overall treated patients and 70% in the RRMS-transplanted patients.^376^ The study also revealed that the benefit of aHSCT on disease progression is greater than that of other high-efficacy immunosuppressive therapies in this type of patient. In this sense, a two-arm randomized clinical trial included 110 RRMS patients who experienced relapses while receiving DMTs and compared disease progression in those who received aHSCT or a DMT of higher efficacy than the one previously used. The authors demonstrated that aHSCT results in a more durable beneficial effect in terms of time to disease progression.^372^ In addition, there is evidence for more sustained disease progression-free survival in SPMS patients treated with aHSCT than in those treated with DMTs.^377^ aHSCT has also been used in clinical practice in patients with aggressive MS as a first-line treatment instead of high-efficacy DMTs. Although there is no consensus on the definition of aggressive MS, the study included 20 patients who presented poor clinical and MRI prognostic features and who underwent transplantation at different centers in Europe and America. The majority of treated patients did not have new inflammatory activity six months after transplantation, and none of them presented new lesions in the following scans. Moreover, all the included patients were disease progression free and presented a significant improvement in disability after aHSCT. This study highlights the high efficacy and durable effect of aHSCT in patients with aggressive MS.^378^

In terms of immune reconstitution, 12 months after HSCT, the naive TCR repertoire is completely renewed, and the reactivity of T cells against MS-related antigens decreases.^379,380^ B-cell numbers recover within three months following HSCT, although the repertoire diversity of memory B cells is deemed one year after transplantation,^381^ and an increased frequency of immune cell populations with an immunomodulatory profile is found in MS-transplanted patients.^376,382^ These are likely the main mechanisms involved in the improvement in clinical and inflammatory activity and could also explain why aHSCT is more effective in RRMS patients who present high inflammatory activity. In contrast, the reactivity toward EBV is increased,^379^ and the lack of diversity of memory B cells increases susceptibility to infections.^381^

In summary, aHSCT has been shown to be highly effective in RRMS patients who do not respond to conventional DMTs, as it suppresses disease activity by eliminating inflammation. In contrast, SPMS patients exhibit a less favorable response to treatment, but its efficacy remains poor in patients with PPMS. The SPMS and PPMS forms of MS are driven primarily by chronic neurodegenerative mechanisms, with limited contributions from acute inflammatory relapses. As such, therapeutic strategies targeting immune-mediated inflammation, including aHSCT, have demonstrated reduced efficacy in these disease forms. While aHSCT has shown high efficacy in suppressing inflammatory activity in RRMS patients, its impact on the slow, progressive neurodegeneration characteristic of SPMS and PPMS remains limited. This distinction in disease biology likely underlies the less effective therapeutic response observed in progressive MS. The European Committee for Treatment and Research on Multiple Sclerosis (ECTRIMS) and the EBMT Autoimmune Diseases Working Party (ADWP) reviewed the current state of aHSCT in adults with MS in 2022 and provided consensus recommendations for the use of aHSCT in patients with MS (see^364^ for extended details). To summarize, the panel of experts agreed that aHSCT is recommended for highly active MS patients refractory to high-efficacy DMTs. In addition, aHSCT can be considered in patients with the aggressive form of MS after the failure of only one high-efficacy DMT. In young individuals ( < 45 years old) with early progressive MS, short disease progression and evidence of inflammatory activity, aHSCT can also be an option. However, there is no evidence to support the use of aHSCT in progressive patients with no inflammatory activity, and it is not recommended to treat MS patients with severe disability and long disease duration because of the high risk and low benefit of this therapy.^364^

## Antigen-specific immunotherapies for MS

The complexity of MS pathogenesis makes it challenging to develop therapies that restore immune self-tolerance without compromising immune function.

Owing to the autoimmune origin of MS, many antigen-specific tolerance approaches are currently aimed at attenuating autoimmunity at the level of peripheral tolerance, and a large proportion of these approaches have already been tested in clinical trials.^383–385^ In this way, we have classified the current antigen-specific immunotherapies for MS into four approaches: peptide- and protein-based, DNA vaccination, peptide-loaded into carrier, and cellular immunotherapy approaches. Table 3 provides a comparative overview of these four approaches, summarizing their key advantages and limitations in the context of MS treatment.

**Table 3:** Comparative overview of antigen-specific immunotherapies in multiple sclerosis

Antigen-specific immunotherapies	Advantages	Disadvantages
**Peptide and protein-based approaches**	High specificity and selectivity in targeting autoreactive immune cells Low toxicity and minimal systemic immunosuppression Versatile mechanisms, including oral tolerance and high-zone tolerance induction	Limited clinical success due to variability in patient response Poor stability and short half-life of peptides High production costs and challenges in delivery
**DNA vaccination approach**	Induces long-lasting antigen-specific immune tolerance Safe and well-tolerated in early trials Broad tolerance induction, extending to multiple myelin antigens Flexible delivery methods (intramuscular, intradermal, gene gun)	Variable efficacy in clinical trials; inconsistent results Potential risk of unintended immune responses Low transfection efficiency compared to viral-based gene delivery Regulatory and standardization challenges
**Peptide-loaded into a carrier approaches**	Enhanced antigen delivery and immune modulation Availability of large-scale manufacturing, low production costs and easy manufacturing processes. Small, soluble and bioavailable carrier platforms Targeted biodistribution	Potential risk of immune activation instead of tolerance Limited large-scale clinical evidence Regulatory challenges due to the novelty of nanotechnology in medicine
**Cellular immunotherapy approaches**	Minimal risk of immune rejection or pro-inflammatory immune response Reduced need for targeted delivery Demonstrated feasibility and safety in preclinical trials	High cost and complex manufacturing of personalized cell therapies Variability in patient response and durability of effects. Limited large-scale clinical validation Ethical and regulatory challenges, particularly with genetically modified cells

*TolDCs* tolerogenic dendritic cells, CAR-Treg cells chimeric antigen receptor T regulatory cells

### Peptide- and protein-based approaches

**Fig. 5:**
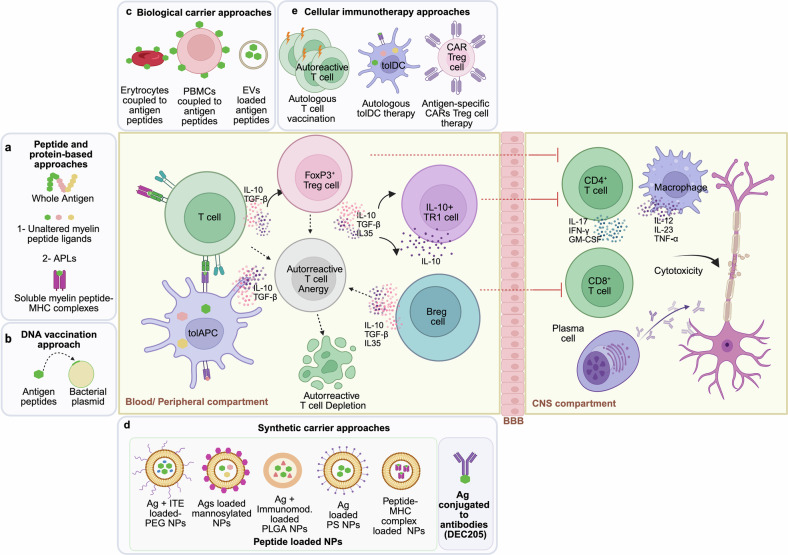
Schematic diagram depicting ongoing antigen-specific immunotherapies for multiple sclerosis. **a** Peptide and protein-based approaches include the use of whole antigens, unaltered myelin peptide ligands, altered peptide ligands (APLs), and soluble myelin peptide–MHC complexes. **b** The DNA vaccination approach relies on a bacterial plasmid carrying a gene encoding a specific antigen peptide. Peptide-loaded carrier approaches can be divided into biological and synthetic methods. **c** Biological carrier approaches induce tolerance by using cells or extracellular vesicles (EVs) as carriers, such as autologous peripheral blood mononuclear cells (PBMCs) or erythrocytes loaded with multiple sclerosis (MS) autoantigen peptides. **d** Synthetic carrier approaches, on the other hand, use drug delivery nanosystems to transport antigenic molecules to target tissues, including pegylated gold (PEG) nanoparticles (NPs), mannosylated NPs, polylactide-coglycolide (PLGA) NPs, and phosphatidylserine (PS) NPs, as well as peptide‒MHC complex-loaded NPs and peptides conjugated to antibodies. **e** Cellular immunotherapy approaches involve immunization with autologous attenuated autoreactive T cells, the use of autologous tolerogenic dendritic cells (tolDCs), and engineered Treg cells expressing myelin-specific chimeric antigen receptors (CARs). These therapies aim to induce an anti-inflammatory microenvironment; promote the secretion of cytokines such as IL-10, TGF-β, and IL-35; induce T-cell anergy; and generate Treg cells, IL-10^+^ regulatory CD4^+^ T-cell type 1 (Tr1) cells, and B regulatory (Breg) cells, ultimately suppressing autoreactive attack and restoring immune tolerance at the central nervous system (CNS) level. Abbreviations: BBB, blood–brain barrier; tolAPC, tolerogenic antigen-presenting cell; Ag, auntoantigen; Immunomod; immunomodulator. Created in https://BioRender.com

A wide range of peptide- and protein-based approaches have been investigated to induce immune tolerance in MS. This perspective includes whole myelin antigens, unaltered myelin peptide ligands, altered myelin peptide ligands, and soluble myelin peptide–MHC complexes (Fig. 5a). These strategies are discussed in more detail below.

#### Tolerization based on the whole myelin antigen

A traditional approach to inducing immune tolerance involves the oral administration of antigens.^386^ Pioneering studies have demonstrated that the oral administration of MBP strongly suppresses EAE in Lewis rats. This MBP-induced oral tolerance is characterized by a reduction in clinical neurological symptoms of EAE, diminished histopathological changes in the CNS, a significant decrease in the specific T-cell proliferative response to the administered antigen, and a decrease in serum antibody levels specific for MBP.^387^ Similarly, in a chronic relapsing model of EAE in B10. In PL mice, the oral administration of MBP either prior to EAE induction or upon the onset of clinical signs leads to a notable decrease in both the frequency and severity of EAE relapse.^388^

Therefore, it is not surprising that the initial endeavour to translate immune tolerance in MS clinical practice revolved around the oral administration of whole bovine myelin, comprising both MBP and proteolipid protein (PLP). The outcomes of a placebo-controlled phase II clinical trial involving 30 RRMS patients who were administered 300 mg of bovine myelin capsules daily for one year revealed that in the myelin-treated group, six out of fifteen individuals experienced at least one major exacerbation of neurological symptoms, compared with twelve out of fifteen in the control group. Moreover, the number of T cells that exhibited reactivity toward MBP was notably lower in the myelin-treated group than in the control group. Importantly, no instances of toxicity or side effects were observed. Nonetheless, the interpretation of these results was hampered by the limited sample size.^389^ In terms of the mechanism of tolerance, oral tolerance is postulated to induce clonal anergy or deletion when an antigen is administered at high doses. Conversely, low-dose administration induces bystander suppression by stimulating Treg cells to release regulatory cytokines, such as transforming growth factor-β (TGF-β), IL-4, or IL-10.^386,390^

#### Tolerization based on unaltered myelin peptide ligands

An alternative strategy for inducing immune tolerance in MS involves the administration of high doses of soluble antigen, known as “high zone tolerance”.^391^ This method involves repeatedly engaging the TCR, leading to programmed cell death of antigen-specific T cells through Fas ligands and TNF-α and potentially inducing unresponsiveness or programmed cell death in antigen-specific B cells.^392–394^ In this context, repetitive intravenous administration of MBP in EAE mice triggers programmed cell death of MBP-specific T cells and alleviates clinical symptoms,^391^ and it also remains effective even after prolonged chronic disease.^395^

In clinical studies, the approach focused on utilizing highly immunodominant myelin peptides, specifically those targeting the MBP_82–98_ region. This region strongly binds to the disease-associated HLA-DR2 molecule (DRB1*1501) and is dominant for MBP-specific T cells in patients with the HLA-DR2 haplotype. Notably, the same residues within the core of this antigen peptide are recognized by MBP-specific autoantibodies, and it contains an additional epitope that can be presented by various HLA-DR molecules, including HLA-DR1, HLA-DR4, HLA-DR7, HLA-DR11 and HLA-DR13.^396^ The underlying hypothesis of this approach posited that the administration of MBP peptides could facilitate tolerance induction in both MBP-specific B cells and T cells. Consequently, a phase I clinical trial was conducted in patients with chronic progressive MS subjected to high doses of the MBP_85–96_ peptide. The results, on the basis of the quantification of MBP-specific autoantibodies in CSF, indicated both tolerability and long-lasting tolerance effects, particularly in patients with disease-associated HLA-DR2.^396^ Notably, the route of peptide administration was crucial, as only intravenous, not intrathecal or subcutaneous, injection induced tolerance to MBP. On the basis of these promising results, a phase II clinical trial was conducted to assess the clinical efficacy of this treatment using MBP_82–98_ in 32 patients with progressive MS, in which changes in the EDSS score were measured. The results revealed the existence of a responder subgroup of SPMS patients carrying the HLA haplotypes DR2 and/or DR4.^397^ This discovery led to the design of a phase III study aimed at evaluating the efficacy and safety of MBP_82–98_ in 612 patients with SPMS, phenotyped as HLA-DR2 or DR4 and an EDSS score of 3.0–6.5.^398^ However, a multicenter randomized two-year, double-blind, placebo-controlled study concluded that, compared with placebo, treatment with MBP_82–98_ did not yield a clinical benefit in the selected population.^398^

In line with this research, a double-blind, placebo-controlled study evaluated the safety and efficacy of a transdermally applied mixture of three myelin peptides (MBP_85–99_, MOG_35–55_, and PLP_139–155_) in 30 RRMS patients. The treatment significantly reduced both MRI-based and clinically defined measures of disease activity, underscoring the safety and tolerability of this approach.^399^ Furthermore, previous studies attributed the inefficient tolerance induction of MBP_89–101_ in EAE to the dominance of a cryptic epitope-presenting MHC conformation.^400^ This underscores the importance of designing therapeutic peptides that mimic natural antigen processing for effective immune modulation in autoimmune diseases.^400^ To achieve this goal, tolerogenic peptides, known as apitopes, are engineered as antigen-processed independent T-cell epitopes. A group of researchers identified four immunodominant epitopes of MBP that exhibited apitope-like behavior. They formulated and tested a cocktail of these peptides known as ATX-MS-1467. Using the (Ob x DR2)F1 mouse model, which expresses the MS-associated HLA-DR2b molecule and a patient-derived TCR specific for the MBP_84–102_ peptide, the peptide cocktail significantly decreased disease severity and delayed onset in both male and female mice in a dose-dependent manner, even when it was administered after disease onset. Importantly, ATX-MS-1467 demonstrated superior therapeutic potential compared with glatiramer acetate.^401^ The phase Ia clinical trial of ATX-MS-1467 demonstrated its safety and good tolerability in a cohort of six patients with SPMS, with a dose of up to 800 µg.^401^ In a subsequent phase Ib study, its safety was further evaluated in 43 patients with relapsing MS, comparing intradermal and subcutaneous administration. The participants received escalating doses of 25, 50, 100, 400, and 800 μg every 14 days over 8 weeks, followed by four additional 800 μg doses at 14-day intervals and a 32-week off-treatment period. A significant reduction in new/persisting Gd-enhanced lesions was observed in the intradermal group at week 16, although values returned to baseline by week 48.^402^ In a subsequent phase IIa single-arm, multicenter trial, 37 participants received an intradermal titration regimen starting at 50 μg on day 1, increasing to 200 μg on day 15 and 800 μg on day 29, followed by biweekly 800 μg doses for 16 weeks, with a subsequent 16-week off-treatment period. Efficacy was assessed through MRI and clinical outcomes, whereas safety endpoints included treatment-emergent adverse events and injection-site reactions. This study revealed a significant reduction in the number and volume of new and total Gd-enhancing lesions in patients with RRMS and a sustained effect posttreatment. However, further clinical trials are needed to confirm the long-lasting therapeutic effects.^402^ In ongoing research, scientists have synthesized the MOG_35–55_ peptide and then transformed its three-dimensional structure into a cyclic form, designated c-MOG_35–55_. Cyclic peptides offer several advantages over linear versions as potential drugs: they are more stable and resistant to breakdown, bind more selectively to target receptors, have a defined structure for easier optimization, and can

serve as templates for creating nonpeptide drugs suitable for oral administration. These combined benefits make them attractive candidates for future drug development.^403^ Notably, immunization combining the c-MOG_35–55_ peptide and MOG_35–55_ in EAE models significantly mitigated clinical disease manifestations and associated pathological aspects, such as demyelination and axonopathy, in both the acute and chronic phases of EAE.^403^ Computational binding and structural analyses revealed that the c-MOG_35–55_ peptide interacts less intensely with mouse or human MHC-II alleles (H2-IAb and HLA-DR2, respectively). Therefore, the milder interactions suggest that cyclic modification weakens the ability of the peptide to activate T cells through MHC-II presentation, potentially leading to a reduced immune response and less severe disease symptoms. These findings provide empirical support for the concept that cyclic modification of a well-established encephalitogenic peptide can have a beneficial effect on clinical outcomes and underlying pathological processes in EAE. This approach to cyclically modifying linear peptides represents a novel therapeutic avenue with potential for future patient-tailored immunomodulatory interventions in the context of MS.^403^

Currently, the investigational compound Neurovax is under examination. It consists of three TCR peptides (BV5S2, BV6S5, and BV13S1) that are overexpressed in myelin-reactive T cells and are associated with MS pathology.^404^ The vaccine works by stimulating Treg cells that specifically target these pathogenic T cells. Upon vaccination, Treg cells produce anti-inflammatory cytokines, including IL-10, which suppresses the activity of disease-causing T cells through bystander suppression. This mechanism not only reduces myelin-specific T-cell responses but also may restore immune tolerance, potentially altering disease progression in MS.^405^ Preclinical studies demonstrated that TCR peptide vaccination with BV5S2, BV6S5, and BV13S1 peptides effectively prevented EAE onset and suppressed disease progression when it was administered after disease induction. The mechanism involved a significant increase in Treg cells and led to a reduction in proinflammatory Th1 cytokines (e.g., IFN-γ) and an increase in anti-inflammatory cytokines, particularly IL-10.^406–408^ The suppression of EAE symptoms was attributed to a bystander suppression effect, where TCR peptide-specific Treg cells not only downregulated the targeted myelin-reactive T cells but also modulated the broader inflammatory response within the CNS. This was evidenced by reduced infiltration of activated T cells into the CNS, decreased demyelination, and lower levels of proinflammatory cytokines in the CSF.^405^ These preclinical findings provide strong proof-of-concept evidence for the immunomodulatory potential of Neurovax, leading to its subsequent evaluation in human clinical trials. In phase I clinical trials, Neurovax exhibited a strong immunogenic response, with a high percentage of participants developing TCR-reactive T cells.^405^ In a double-blind, placebo-controlled study, 55% of participants who responded to the vaccine showed a significant reduction in the number of circulating myelin-reactive T cells, and none experienced clinical worsening, in contrast to 59% of nonresponders or placebo recipients who exhibited disease progression. The vaccine was well tolerated, with the most common adverse events being mild injection-site reactions.^405,409,410^ Despite its therapeutic potential, its clinical trials have faced repeated delays, with no recent updates on its progress. Three planned studies include a phase IIb trial in SPMS (150 participants, assessing disability progression over 48 weeks), a phase II trial in SPMS (200 participants, evaluating new active lesions on MRI), and a phase I trial in pediatric MS (12 participants, assessing safety and efficacy). However, the current status of these trials remains unclear, raising concerns about the feasibility and timeline of Neurovax’s development (NCT02200718, NCT02149706, and NCT02057159).

In summary, clinical trials involving peptide-based therapies have yielded diverse outcomes, which may be attributed to variations in the mode of administration, patient cohorts, and the use of single- versus multipeptide treatments.

#### Tolerization based on altered myelin peptide ligands

Altered peptide ligands (APLs) are modified versions of natural peptides that influence T-cell function by either blocking activation (antagonists) or inducing weak stimulation (partial agonists). Their ability to modulate immune responses opens new therapeutic venues, where copresentation with the original peptide could suppress harmful immune activity by altering T-cell signaling. and reducing their effector functions.^411^

In this sense, some preclinical studies explored APLs for modulating T-cell responses in MS. The first preclinical approach involved [A^4^]MBP_1–11_ with an alanine substitution that enhanced MHC-II binding and prevented EAE.^412^ In another study, researchers investigated the MBP_72–85_ peptide, which is crucial for the immune response in Lewis rats. They modified three specific TCR contact residues (Lys^73^, Arg^76^ and Asp^79^), but none of these modifications had therapeutic effects on EAE.^413^ A cyclic version of the MBP_72–85_ peptide, known as cyclo(75–82)MBP_72–85,_ combined with a specific variant containing an alanine substitution completely prevented EAE.^414^ Similarly, [A^91^]MBP_87–99_ suppressed EAE by inhibiting the production of TNF-α and IFN-γ via either linear or cyclic APLs from MBP_87–99_.^415^ Notably, cyclo(87–99)[R^91^,A^96^]MBP_87–99_ completely blocked EAE development, whereas cyclo(91–99)[A^96^]MBP_87–99_ had weaker tolerogenic effects.^416,417^ In addition, researchers have explored another APL derived from a PLP peptide [L^144^, R^147^]PLP_139–151_. Preimmunization with this specific variant offered protection against EAE even when triggered with other unrelated myelin antigens. This observation implies that the peptide might not directly block specific T cells (acting as an antagonist) in vivo but might work through bystander suppression, potentially triggering the generation of Treg cells that can suppress various harmful immune responses, offering broader protection beyond the targeted antigen.^418^ Similarly, another research group focused on mutating the TCR contact sites of MBP_83–99_ peptides and conjugating them to reduced mannan, a mannose-containing polysaccharide, which has immunomodulatory properties. In this study, MBP_83–99_ peptides conjugated to reduced mannan led to a shift toward a Th2 immune response and to the production of antibodies that did not cross-react with the native MBP protein in a mouse model of EAE.^419^ Additionally, the same researchers synthesized cyclic peptides by mutating the TCR contact sites of the MBP_83–99_ epitope, aiming to overcome the limited stability of linear peptides, and then conjugated these peptides with reduced mannan. Compared with those of the native peptide, the preclinical results demonstrated enhanced IL-4 responses.^420^ These findings suggest the potential of APLs for MS therapy. More recently, a new study investigated the efficacy of amide-linked cyclic peptide analogs of MBP_87–99_, which are mutated at positions 91 and/or 96, as both prophylactic and therapeutic protection against acute EAE.^421,422^

The first APL tested in clinical trials was derived from the MBP_83–99_ peptide and was named CGP77116. It was evaluated in patients with RRMS who received weekly subcutaneous injections during a phase II clinical trial. However, the study had to be prematurely halted owing to exacerbations observed in three out of eight patients who received high doses of CGP77116. These exacerbations are associated with the appearance of new inflammatory lesions on MRI scans and are correlated with a significant increase in the number of encephalitogenic MBP_83–99_-reactive T cells.^423^ A larger placebo-controlled multicenter trial involving 142 RRMS patients was subsequently conducted. Patients were treated with weekly subcutaneous injections of either placebo or an APL known as NBI-5788, which was also derived from the MBP_83–99_ peptide. Unfortunately, this trial was also halted because hypersensitivity reactions were observed in 9% of the patients. Patients experienced stabilization of clinical relapses and the absence of new Gd-enhancing lesions; however, the majority of patients developed Th2-driven hypersensitivity reactions after receiving more than 10 doses of NBI-5788. Interestingly, APLs elicit a regulatory Th2 response that cross-reacts with the native peptide, suggesting potential immunomodulatory effects beyond its intended target.^424^ A secondary analysis of the patients who completed the study revealed that both the volume and the number of gadolinium-enhancing lesions were reduced when the drug was administered at a 5 mg dose.^424^ The authors concluded that APLs could represent a novel class of treatments for MS, but the reported side effects led them to state that the potency of the Th2 response must be controlled.^424^ However, since then, no new clinical studies involving APLs have been initiated.

#### Tolerization based on soluble myelin peptide-MHC complexes

An alternative approach to induce immunotolerance in MS involves the utilization of myelin peptides loaded on MHC complexes (pMHCs). These complexes interact specifically with TCR ligands in the absence of costimulatory signals,^425^ leading to the induction of T-cell anergy and/or deletion, thereby preventing the activation of autoreactive T cells.^426^ Initial investigations focused on the evaluation of soluble MHC-II molecules complexed with PLP_139–151_ or MBP_91–103_ in EAE.^427^ The results revealed a significant reduction in the severity and progression of EAE symptoms in SJL mice treated with soluble complexes (MHC-II molecules (I-A^s^) complexed with PLP_139–151_ or with MBP_91–103_) compared with untreated controls, suggesting their potential as a therapeutic strategy for MS.^427^ The encouraging outcomes from these preclinical studies prompted the initiation of a phase I clinical trial in 2000, which enrolled 33 patients afflicted with SPMS who were HLA-DR2 positive. Patients received intravenous administration of HLA-DR2/MBP_84–102_ or a placebo. While the trial established the safety and tolerability of this approach, it did not reveal any clinical or radiological efficacy.^428^ A group of researchers subsequently developed recombinant TCR ligands (RTLs) with the same HLA-DR2 haplotype and pMHC-TCR interaction framework. RTLs consist of a single polypeptide chain comprising the β1- and α1-domains of MHC-II molecules coupled with an autoantigenic peptide. The rationale underlying this approach lies in the augmented production of regulatory cytokines by T cells, attributed to the absence of the β2-domain of MHC-II molecules, which typically houses the CD4 coreceptor binding site.^429^ EAE models have demonstrated that RTL therapy effectively prevents and reverses EAE pathology, diminishes the severity of CNS lesions, and promotes neuronal regeneration.^430–433^ In 2012, a phase I dose-escalation study involving a cohort of 34 HLA-DR2 MS patients was conducted. These patients were treated intravenously with the two outer domains of HLA-DR2 coupled to MOG_35–55_ (known as RTL1000) to ascertain the maximum tolerated dose, safety, and tolerability.^434^ The study revealed that RTL1000 was well tolerated and safe at doses up to 60 mg. Notably, there was no evidence of increased disease activity on the basis of MRI. However, owing to the limited number of patients in this trial, no definitive conclusions regarding efficacy could be drawn.^434^ These findings underscore the potential of pMHC-based immunotolerance strategies in the context of MS treatment, although further research and larger-scale clinical trials are needed to establish their therapeutic efficacy.

Taken together, peptide- and protein-based therapies hold great promise in precision medicine because of their high specificity, selectivity, and ability to target diverse biological pathways with minimal toxicity. The rapid development and engineering flexibility of these materials further increase their therapeutic appeal. However, their clinical translation faces significant challenges, including poor stability, short half-life, and inefficient delivery, as they often exhibit low oral bioavailability and struggle to cross biological barriers. Additionally, high production costs, potential immunogenicity, and stringent storage requirements further complicate their widespread adoption. Future progress hinges on the integration of innovative formulation technologies, advanced delivery systems, and rational design approaches to optimize their therapeutic potential and establish them as viable mainstream treatments.

### DNA vaccination approach

DNA vaccination is a promising approach for the treatment of MS and was proposed as early as 1996 by Waisman and colleagues.^435^ DNA vaccines utilize bacterial plasmids carrying a gene encoding the desired antigen(s) controlled by a mammalian promoter. Additionally, the presence of unmethylated cytosine‒phosphate‒guanosine (CpG) motifs within the plasmid structure further stimulates the immune response.^435^ Once delivered, various cell types take up plasmids, express the encoded genes, process antigens, and present them to immune cells, thereby initiating a specific immune response or altering an existing response (Fig. 5b). DNA vaccines are often delivered subcutaneously, intradermally or intramuscularly, with the dermis and muscle being the principal transfected tissues. In skeletal muscle, myocytes primarily take up plasmids, while a few APCs may also be transfected. Myocytes can present antigens to T cells directly or produce antigens that are then picked up by APCs for presentation. The gene gun delivery system uses tungsten or gold particles coated with gene-encoding DNA plasmids that are propelled to the target tissue, allowing more efficient delivery of the DNA vaccine into target cells. This technology allows the transfection of DNA plasmids directly into myocytes and, to a smaller degree, into resident APCs in the skin.^436^ Animal studies suggest that DNA vaccination triggers different tolerance mechanisms depending on the immunization route. Transfected myocytes may induce T-cell anergy due to the lack of costimulatory molecules, leading to reduced T-cell activation. Additionally, intramuscular DNA vaccination induces IFN-β production via TLR9 activation, downregulating IL-12 expression and diminishing Th1 and Th17 responses. Intradermal vaccination with a gene gun induces protective Th2 immune responses characterized by the secretion of regulatory cytokines such as IL-4, IL-10, and TGF-β. These findings shed light on the diverse mechanisms underlying DNA vaccination and its potential for modulating immune responses in autoimmune diseases such as MS.^436^ Since 1997, many studies have been published on EAE models in which DNA vaccination has been performed and various myelin antigens and different immunomodulatory sequences have been evaluated with varying degrees of success.^437–443^

A DNA vaccine containing the MBP gene prevented EAE in eight out of nine Lewis rats. Protection correlated with MBP-specific IgG1 antibodies, indicating a Th2-driven immune response and supporting the role of Th2 immunity in EAE suppression and recovery.^437^ In another study, researchers utilized DNA containing a minigene encoding PLP_139–151_. This suppressive vaccination strategy effectively mitigated EAE, reducing proliferative responses and diminishing the production of Th1 cytokines, including IL-2 and IFN-γ, thus decreasing brain mRNA levels of proinflammatory cytokines. Mechanistically, the reduction in disease severity and cytokine production was linked to altered T-cell costimulation, resulting in impaired T-cell proliferation, even in the presence of CD28 costimulation, as well as altered expression of CD80 and CD86 on APCs in the spleen.^438^ Immune cell phenotypes were studied following DNA vaccination with MOG_91–108_ and subsequent EAE induction. Among the rats protected from EAE, no changes in antigen-specific Th2 or Th3 responses were detected. However, MHC-II expression on splenocytes decreases early after treatment, antigen-specific IFN-β production is elevated upon recall stimulation, and lymphocytes present reduced IL-12β2 receptor levels.^440^ In a previous work, researchers explored the impact of coadministering plasmid DNA vaccines encoding IL-10 alongside a plasmid encoding MBP_68–86_ during active disease. The combined treatment rapidly enhanced the Tr1 cell-mediated response, specifically in the context of ongoing disease. Administration of both plasmids together, but not the MBP plasmid alone, swiftly suppressed ongoing disease, leading to increased IL-10-producing antigen-specific T cells and increased apoptosis of cells around high endothelial venules in the CNS after therapy. Transfer of tolerance was achievable via MBP-specific primary T cells from protected donors, and reversal was observed with neutralizing antibodies to IL-10 but not IL-4. This study suggested that effective induction of tolerance depends on redirecting the Tr1 cell response to the dominant epitope during a given phase of the disease.^442^

This approach led to phase I/II and phase II clinical trials. The first was a randomized, double-blind, placebo-controlled trial of BHT-3009, a tolerizing DNA vaccine encoding full-length human MBP. The study design included a total of 30 RRMS and SPMS patients enrolled in placebo, BHT-3009 alone, and BHT-3009 plus atorvastatin calcium and involved intramuscular administration of the DNA vaccine at weeks 1, 3, 5, and 9 after randomization. The authors demonstrated that BHT-3009 was safe and well tolerated. In addition, the results revealed an induction of tolerance to the antigen-specific autoreactive immune response with a favorable reduction in inflammatory lesions, downregulation of IFN-γ-producing CD4^+^ T cells in the peripheral blood, and a decrease in myelin-specific antibody titers in the CSF, indicating a low level of myelin-specific immune responses in both the periphery and the CNS. Tolerance induction is not limited to MBP peptides but extends to other myelin proteins, including PLP, MOG, and αB-crystallin.^444^ During the phase II clinical trial, the therapeutic efficacy of BHT-3009 was assessed in a cohort of 289 patients diagnosed with RRMS. These patients were randomly assigned to one of three groups: a placebo group, a group receiving 0.5 mg of BHT-3009 intramuscularly, and a group receiving 1.5 mg of BHT-3009 intramuscularly. The study outcomes revealed that the administration of the 0.5 mg dose of BHT-3009 led to a significant reduction in the frequency of MRI-detected lesions. Additionally, this treatment regimen is associated with the induction of antigen-specific immune tolerance.^445^

Overall, this approach employs versatile delivery methods (intramuscular, intradermal, or gene gun) to induce immune tolerance via T-cell anergy and Th2 bias. Clinical trials have confirmed the safety, tolerability, and reduced incidence of inflammatory lesions, with plasmid-based immunomodulation enhancing Treg cell responses. However, translation remains limited, with no trials beyond phase II and uncertain long-term efficacy. Route dependency, CpG-driven innate activation, and preclinical inconsistencies highlight challenges in optimizing multiplasmid formulations. Species-specific differences and HLA-independent mechanisms further limit broad applicability.

DNA vaccination offers a precise and adaptable strategy for MS, but gaps in clinical validation, route optimization, and mechanistic clarity persist. Future efforts should refine plasmid design, incorporate biomarker-driven dosing, and explore adjunct therapies to increase regulatory responses. While promising, success hinges on overcoming translational hurdles and demonstrating sustained efficacy in larger, diverse cohorts.

### Peptide-loaded carrier approaches

Recognizing the limitations encountered in clinical trials involving peptide antigens for tolerance induction, including variability in immune responses, inefficient APC transfection, and challenges translating animal model success to humans, alternative therapeutic strategies have explored the use of cells, extracellular vesicles (EVs) and nanoparticles as antigen delivery vehicles. These innovations draw inspiration from the natural capacity of DCs, apoptotic cells, and cell bodies to safeguard against inflammation and sustain self-tolerance within physiological contexts.^446^ The following sections provide a more comprehensive examination of these delivery systems.

#### Biological carrier approaches

This approach uses autologous PBMCs, erythrocytes, and EVs loaded with MS autoantigen peptides as carriers to induce tolerance.^446,447^ Among the advantages of these strategies, the use of autologous cells crosslinked to autoantigens appears to be well tolerated at all stages of disease and greatly reduces the risk of anaphylactic reactions.^448^ Fixation of autoantigens on PBMC supports is possible because of the presence of a chemical crosslinker known as 1-ethyl-3-(3-dimethylaminopropyl)carbodiimide (ECDI)^446^ (Fig. 5c). The first preclinical experiments in EAE models revealed that a single intravenous administration of ECDI-fixed splenocytes loaded with MSCH (a complex mixture of neuroantigens) or with purified PLP resulted in significant protection against EAE development.^449^ In addition, the intravenous injection of splenocytes coupled with PLP_139–151_, PLP_178–191_, or MBP_84–104_ induced myelin-specific tolerance to relapse-associated epitopes in a relapsing-remitting EAE model, effectively preventing initial disease relapse before disease onset and subsequent relapse in SJL mice immunized with PLP_139–151_ or MBP_84–104_.^450^ More importantly, several authors have attempted to target multiple antigenic epitopes to overcome the phenomenon of epitope spreading. In this context, splenocytes coupled with a peptide cocktail of different epitopes, including PLP_139–151_, PLP_178–191_, MBP_84–104_, and MOG_92–106_, were administered at the disease peak to PLP-immunized SJL mice. The treatment inhibited the occurrence of EAE symptoms induced by any single peptide and by a mixture of all the mentioned peptides. This effect is correlated with the inhibition of autoreactive Th1 cells and the consequent infiltration of inflammatory cells into the CNS.^451^

The first clinical trial demonstrated the feasibility, favorable safety and good tolerability of a single infusion of autologous PBMCs loaded with seven myelin peptides (MOG_1–20_, MOG_35–55_, MBP_13–32_, MBP_83–99_, MBP_111–129_, MBP_146–170_, and PLP_139–154_) in seven RRMS and two SPMS patients. Moreover, higher doses of this treatment (1 × 10^9^ antigen-coupled cells infused) resulted in a lower antigen-specific T-cell response.^452^ The crosslinking of peptides into cells induces their apoptosis, and those apoptotic antigen-loaded cells are engulfed by splenic marginal zone macrophages, which present the autoantigen of interest in a tolerogenic manner and consequently produce IL-10 and upregulate the inhibitory molecule PD-1 ligand. On the other hand, apoptotic cells carrying the autoantigen also induce the activation of a Treg-cell subset, which seems to be responsible for long-term maintenance of tolerance.^453^ Similarly, other groups have exploited the potential of erythrocytes as carriers of antigen peptides in MS. The central goal here is to induce tolerance through the capacity of erythrocytes to undergo eryptosis, which is a physiological mechanism of apoptosis that maintains homeostasis and the number of erythrocytes.^454^ After characteristic eryptotic cell shrinkage, the loss of Cl^-^ ions leads to the release of prostaglandin E2, an increase in Ca^2+^ ion levels and a phospholipid shift in the cell membrane. All of these physiological changes result in phosphatidylserine (PS) exposure on the cell surface, which is recognized and engulfed by circulating macrophages with specialized PS receptors to ensure erythrocyte removal from the circulation and avoid the presentation of self-antigens in a proinflammatory context.^455^ In EAE studies, mice were treated on days 0, 7 and 14 postimmunization (p.i.) with erythrocytes coupled to MOG_35–55_ peptides. Coupling of peptide-containing cells was performed with a specific human antibody fragment library (Fab). EAE progression decreased after day 10 p.i. and demonstrated the induction of pathogenic T-cell dysfunction. This “self-tolerance” state is characterized by increased expression of specific molecules such as PD-1 and CTLA-4, which typically suppress immune responses. Remarkably, this effect persisted even months after the initial antigen exposure, suggesting long-lasting immune reprogramming.^456^ Similar studies have been carried out in EAE models, with successful results in alleviating and even preventing EAE outcomes. Specifically, the administration of erythrocytes linked to MOG_35–55_ to C57BL/6 mice before EAE induction with the same peptide delayed the onset or completely suppressed EAE. Histological examination of the spinal cord revealed a lack of both infiltrating inflammatory and Treg cells, suggesting a potential therapeutic effect of erythrocytes carrying MOG_35–55_ in reversing early inflammation and clinical signs of EAE.^457^

A multicenter, phase Ib/IIa clinical trial to evaluate the safety and efficacy of autologous peptide-coupled red blood cells, designated CLS12311, in patients diagnosed with RRMS started in 2023. This study primarily evaluated the safety and tolerability of CLS12311 while analyzing its efficacy in reducing the number of new lesions as a measure of inflammatory disease activity in patients with RRMS (MSB-IG-H-2101).

While cell carrier approaches hold promise for antigen-specific immunotherapies, they encounter several limitations, including variability in the lifespan and migratory capacity of carrier cells, challenges in achieving targeted antigen delivery, potential immunogenicity triggering unwanted immune responses, scalability and cost-effectiveness issues, and regulatory complexities.^458^ Biocompatible solutions, including EVs, have been investigated to overcome these limitations, with additional strategies outlined in the upcoming sections.

A recent study highlighted the critical role of MHC-II-mediated presentation of endogenous “guardian” peptides in maintaining immune tolerance within the CNS under homeostatic conditions^220^; however, during acute neuroinflammation, this protective mechanism is disrupted. To harness this natural regulatory pathway, EVs were engineered to deliver MBP_160–175_, as part of one of the endogenous peptides that were identified to maintain CNS tolerance, into the CSF of EAE-induced mice.^220,459^ Presymptomatic administration of EV-encapsulated MBP_160–175_ significantly suppressed disease severity compared with that in controls receiving citrullinated MBP_160–175_, nonregulatory MBP_192–216_, or irrelevant OVA_323–339_ peptides. Mechanistically, EV-mediated delivery expanded a unique CTLA-4^+^FoxP3^−^CD4^+^ suppressor T-cell population in the dura and draining lymph nodes, whereas conventional Treg cells remained unaffected.^220^ Single-cell RNA sequencing revealed clonal expansion of the CTLA-4^+^FoxP3^−^CD4^+^ suppressor T-cell subset, which displayed a suppressive transcriptional signature marked by elevated *Ctla4*, *Tgfb1*, and phosphatase regulators (*Ptpn6*, *Ptpn11*). Crucially, free MBP_160–175_ peptides failed to replicate this therapeutic effect, underscoring the necessity of EV-based targeted delivery.^220^ These findings position EV-driven antigen-specific immunotherapy as a promising strategy against neuroinflammatory disorders. By leveraging endogenous regulatory peptides, EV platforms selectively amplify CNS-protective immune responses, offering a precision tool to modulate autoreactive T-cell activity.^220^

Despite the promising therapeutic potential of EV-based therapies, their clinical translation is hindered by significant limitations, including inherent heterogeneity, inconsistent isolation and characterization, and potential immunogenicity.^460^ However, EVs offer distinct advantages over cell carrier therapies; mitigate the risks of gene mutation, uncontrolled cell division, and immune rejection; and demonstrate superior stability, biocompatibility, and tissue penetration.^460^ These features have fuelled significant interest in EV-based therapeutics. Nevertheless, to fully realize this potential and address the current challenges, researchers are actively exploring biocompatible alternatives, as detailed in the subsequent sections. These efforts aim to refine EV-based therapies and overcome the obstacles currently impeding their widespread application.

#### Synthetic carrier approaches

Nanotechnology has emerged as an alternative solution to overcome the difficulties associated with biological carriers. Indeed, drug delivery nanosystems are considered novel technology platforms that can transport antigenic molecules to target tissues while increasing therapeutic effectiveness and reducing potential negative effects^461^ (Fig. 5d). Thus, some researchers have focused on the fabrication of artificial vesicles or lipid nanocarriers, such as liposomes or nanoparticles.^462^ In depth, nanoparticles consist of solid colloidal particles made of polymers or lipids and typically range in size from 10 to 1000 nm, with the most common range falling between 50 and 300 nm. These particles serve as carriers for drugs, either by embedding them within a matrix or depositing them on the surface, enabling targeted delivery to specific body parts. For effective drug delivery, nanoparticles must possess certain properties: they should be nontoxic, biodegradable, and biocompatible; exhibit stability in the bloodstream without undergoing aggregation reactions; evade uptake by the mononuclear phagocytic system to ensure prolonged circulation in the blood; enable efficient delivery across the BBB through receptor-mediated endocytosis by brain capillary endothelial cells; be capable of transporting small molecules, peptides, proteins, or nucleotides; cause minimal changes to the drug, such as chemical degradation or structural alterations due to the nanoparticle excipient; offer controllable drug release; and allow for cost-effective and efficient production processes.^463^ Furthermore, nanoparticles may be easily surface modified with functional biomaterials, allowing them to gain enhanced properties.^464^ Owing to their small size, nanoparticles are easily ingested by cells, making them ideal carriers for antigen delivery. Similarly, antigen-loaded nanoparticles offer numerous benefits for antigen-specific immunomodulation, including sustained antigen release, antigen and adjuvant codelivery, antigen depot formation at the injection site, effective presentation of B-cell epitopes, and increased uptake and stimulation of cell-mediated immune responses against acellular antigens.^465^ The encapsulation of certain antigenic myelin peptides in nanoparticles led to immunomodulation of T-cell activity and stimulation of Treg cell and DC development, thereby restoring immunological tolerance.^466^

In addition to polymer or lipid nanoparticles, gold nanoparticles are inorganic nanomaterials known for their exceptional stability and rigidity, maintaining their integrity even at low concentrations. Unlike organic nanomaterials, gold nanoparticles provide a robust platform for drug delivery and are capable of chemically or physically loading drug molecules onto their surfaces. The uptake of gold nanoparticles by cells depends on factors such as size, shape, surface charge, and ligand functionality. Typically, ranging from 10 to 100 nm in size, gold nanoparticles exhibit size- and shape-dependent half-lives in the bloodstream. Furthermore, polymer coatings can be added to gold nanoparticles to increase their drug-loading efficiency, circulation time, biodistribution, and overall functionality. When conjugated with drugs via electrostatic interactions or various linkers, such as thiols, amides, or hydrazine, gold nanoparticles serve as highly effective drug carriers. To bypass the reticuloendothelial system, the main barrier in systemic delivery, polyethylene glycol is frequently used to absorb water molecules, thus enabling gold nanoparticles to evade macrophage detection, thereby preventing their removal and reducing nonspecific binding during delivery while improving the efficiency of drug delivery to target cells.^467^

##### Peptide-loaded nanoparticles

Some scientists have proposed that PEGylated gold nanoparticles, which carry both a disease-relevant autoantigen and a ligand-activated transcription factor known as the aryl hydrocarbon receptor (AhR), might stimulate the generation of antigen FoxP3^+^ Treg cells. This hypothesis stems from previous research indicating that AhR plays a crucial role in regulating the differentiation of FoxP3^+^ and IL-10^+^ Treg cells, as well as Th17 cells, in both mice and humans.^468–470^ The activation of AhR via the mucosal ligand 2-(1′H-indole-3′-carbonyl)-thiazole-4-carboxylic acid methyl ester (ITE) has been demonstrated to expand Treg cells and alleviate EAE.^470^ Additionally, subsequent studies have shown that AhR ligands induce the generation of tolerogenic DCs, which, in turn, promote the differentiation of FoxP3^+^ Treg cells.^471–473^ In preclinical studies in C57BL/6 EAE model mice immunized with MOG_35–55_ and treated with gold nanoparticles carrying both ITE and MOG_35–55_, Treg cell generation increased, and the progression of the experimental disease was inhibited.^474^ Furthermore, the same authors conducted another experiment in which SJL EAE mice were immunized with PLP_139–151_ and treated on day 17 p.i. with gold nanoparticles loaded with ITE and PLP_139–151_, ITE and PLP_178–191_, or ITE and both PLP epitopes. The administration of gold nanoparticle-loaded ITE + PLP_139–151_ transiently improved EAE progression, whereas gold nanoparticle-loaded ITE + PLP_178–191_ significantly reduced EAE severity, the maximum EAE score, and relapse frequency after treatment initiation. These findings suggest that combining gold nanoparticles with multiple relevant T-cell reactivities can effectively modulate epitope spreading and chronic inflammation in established CNS autoimmunity.^474^

According to some studies, encapsulating immunodominant peptides in their native form within mannosylated liposomes offers a significant advantage. This encapsulation, coupled with the presence of mannose residues on the liposome surface, enables targeted delivery to APCs. These cells are equipped with a greater density of mannose receptors, such as the CD206 receptor in DCs, facilitating the efficient endocytosis of mannosylated liposome particles.^475^ The antigen presentation efficiency in the mannosylated form can be up to 10,000 times greater than that in the native form.^476,477^ In this context, a preclinical study used three immunodominant peptides derived from MBP encapsulated in mannosylated small unilamellar vesicles to treat EAE in Dark Agouti rats. Liposome-encapsulated MBP_46–62_ was the most effective at reducing the maximum disease score during the initial attack. Moreover, MBP_124–139_ and MBP_147–170_ completely prevented the development of the exacerbation stage. Both the mannosylation of liposomes and the encapsulation of peptides are crucial for therapeutic efficacy. Nonmannosylated liposomes, whether loaded or empty, did not demonstrate effectiveness. The liposome-mediated delivery of the mixture of three MBP peptides significantly suppressed EAE progression. Furthermore, it prevents the production of circulating autoantibodies, downregulates the synthesis of Th1 cytokines, and induces the production of brain-derived neurotrophic factors in the CNS. Thus, this proposed formulation ameliorated EAE, resulting in a less severe initial attack and facilitating rapid recovery from exacerbation, suggesting a promising therapeutic approach for the treatment of MS.^478^ In this way, a first-in-human, dose-escalation study evaluated the safety of CD206-targeted liposomal delivery of coencapsulated immunodominant MBP_46–62_, MBP_124–139_, and MBP_147–170_ (Xemys) in the treatment of 20 patients with RRMS or SPMS refractory to first-line DMTs. Those patients received weekly subcutaneous injections of Xemys at escalating dosages up to a total dose of 2.675 mg. Clinical evaluation was performed up to 17 weeks after the final treatment. The results revealed the safety and good tolerance of the formulation when it was administered for six weeks at doses ranging from 50 μg to 900 μg. Compared with those at baseline, the numbers of T2-weighted and new Gd-enhancing lesions on MRI did not differ at the end of the study; however, the number of active Gd-enhancing lesions at weeks 7 and 10 increased in comparison with baseline. During the treatment period, the blood levels of the cytokines monocyte chemoattractant protein-1 (MCP-1), macrophage inflammatory protein-1 (MIP-1), and IL-7 were reduced, whereas the level of TNF-α was increased.^479^

Other researchers aimed to induce antigen-specific immune tolerance and developed polylactide-coglycolide (PLGA) nanoparticles that encapsulate peptide antigens alongside the tolerogenic immunomodulator rapamycin. This approach triggers the generation of regulatory DCs while inhibiting the mTOR pathway. The results of EAE models revealed protection from relapse due to the inhibition of antigen-specific CD4^+^ and CD8^+^ T cells and B cells while the induction of antigen-specific Treg and B regulatory (Breg) cells.^480^ C57BL/6 MOG_35–55_-induced EAE mice were prophylactically and therapeutically treated with PLGA nanoparticles loaded with the MOG_35–55_ peptide. Prophylactic administration (on day 7 before EAE induction) of MOG-conjugated PLGA nanoparticles demonstrated the most promising results in this mouse model of MS, significantly reducing disease severity and brain inflammation and promoting a regulatory immune response. Nonetheless, the therapy also proved to be efficient when it was administered to already diseased EAE mice.^481^ In another effort to improve the interaction with DC mannose receptors, PLGA nanoparticles loaded with the MOG_35–55_ peptide conjugated with saccharide residues such as glucosamine have recently been developed. Preclinical data demonstrate the potential of glycosylated peptides encapsulated in PLGA nanoparticles as therapeutic vaccines in an EAE model. C57BL/6 EAE mice treated with PLGA nanoparticles loaded with glycosylated MOG_35–55_ showed significant suppression of disease symptoms. Interestingly, those treated with glycosylated MOG_35–55_ experienced immediate remission after the second dose, with relapse occurring later, suggesting that the continuous release of glycosylated MOG_35–55_ is necessary for sustained immune suppression, possibly through interference with DC antigen presentation.^482^ In additional EAE studies, another strategy involving the coencapsulation of the immunomodulator dexamethasone with MOG_35–55_ into acetylated dextran microparticles led to considerable improvements in clinical outcomes.^483^ Similarly, the efficacy of PLGA microparticles conjugated with peptide antigens and TGF-β was superior to that of peptide-loaded microparticles alone in EAE mice.^484^ A novel and innovative nanoparticle-based approach for inducing immunological tolerance has recently emerged. This approach involves the development of a dual peptide-loaded nanoparticle platform designed to modulate the immune response. The dual platform comprises PLGA nanoparticles loaded with two types of peptides: an antigenic peptide serving as the primary signal and an inhibitory peptide targeting the costimulatory signal. Specifically, PLGA nanoparticles are encapsulated with an ICAM-1-binding peptide, which effectively inhibits the costimulatory signal involved in T-cell activation. Additionally, these nanoparticles are conjugated on their surface with MOG_35–55_ peptides. The dual peptide nanoparticles were administered intravenously either prophylactically or therapeutically to MOG_35–55_ C57BL/6-immunized mice. In the prophylactic approach, the treatment completely prevented the occurrence of EAE, whereas both the prophylactic and therapeutic approaches decreased inflammatory cell infiltration and demyelination in the spinal cord. In particular, in therapeutic experiments, the dual peptide nanoparticles had a stronger inhibitory effect on EAE than the MOG peptide nanoparticles alone did. Mechanistically, the dual peptide nanoparticles reduced the expression of MHC-II and the costimulatory molecule CD86 on the surface of DCs, inducing abortive T-cell activation. This ultimately resulted in decreased infiltration of Th1 and Th17 cells in the CNS and demonstrated antigen-specific immune tolerance. Thus, dual peptide nanoparticles hold significant potential for the treatment of MS by inducing immune tolerance.^485^ Another group was inspired by the capacity of the coinhibitory receptor B and T lymphocyte attenuator (BTLA) to regulate peripheral tolerance. Accordingly, a nanoparticle encapsulating the MOG_35–55_ peptide with BTLA was engineered and loaded into DCs. Treatment of MOG_35–55_-induced EAE mice increased Treg generation and IL-10 and TGF-β secretion. In addition, immunotherapy suppressed the CD4^+^ T-cell response to MOG and inflammation in the spinal cord and reduced disease severity, while the immune response to irrelevant antigens was not modified.^486^

As previously mentioned, several research initiatives have drawn inspiration from the physiological process of tissue turnover, in which useless cells undergo apoptosis and are rapidly cleared. To take advantage of this naturally occurring tolerance, liposomes mimicking apoptotic bodies have been proposed as carriers of self-antigens to induce antigen-specific tolerance in the context of autoimmunity. Liposomes are versatile lipid-based vesicles that can be engineered to display PS molecules on their surface. As mentioned earlier, PS phospholipids are exposed on the outer leaflet of the membrane when the cell enters apoptosis.^487^ The externalization of PS serves as an “eat me” signal for clearance and “tolerates me” signal for immune tolerance to self-antigens. This unique feature enables loaded antigens to be presented in a tolerogenic manner.^488^ On this basis, some researchers have postulated that PS nanoparticles carrying autoantigens might elicit tolerogenic immune responses (109). In line with this concept, PS liposomes loaded with the MOG_35–55_ antigen were tested with the aim of inducing antigen-specific tolerance in the MOG-induced EAE model. PS liposomes induce a tolerogenic phenotype in DCs and significantly reduce the incidence and severity of the experimental disease.^488^ This approach was further adapted to address other autoimmune disorders by simply substituting the encapsulated autoantigen. Conditions such as type 1 diabetes, rheumatoid arthritis, and myasthenia gravis could benefit from this adaptable and innovative approach.^489^

Expanding on this concept of peptide-loaded nanoparticles, another promising strategy involves the use of peptide-MHC (pMHC) complex-loaded nanoparticles to directly modulate autoreactive T-cell responses. This approach is based on the hypothesis that the engagement of the TCR with its pMHC target without costimulation results in the induction of apoptosis or anergy. Furthermore, this approach proposes that pMHC-loaded nanoparticles hold promise as a more effective strategy to control T-cell activity in autoimmune diseases than traditional approaches using soluble pMHC molecules. The potential benefits lie in the ability of nanoparticles to interact with multiple T cells simultaneously, potentially triggering a specific suppressive response, and offering protection from degradation, leading to a more sustained effect.^426^ In this context, the potential of nanoparticles coated with MOG_38–49_/IAb to mitigate the progression of MOG_35–55_-induced EAE in C57BL/6 mice was investigated. Administration of MOG_38–49_/IAb nanoparticles therapeutically on day 14 p.i. attenuated disease progression and restored motor function when administered on day 21 p.i. These therapeutic effects were accompanied by weight gain and correlated with a systemic expansion of antigen-specific Tr1 cells, a reduction in activated macrophages/microglia in the cerebellum, decreased inflammatory foci and areas of demyelination in the white matter of the cerebellum, and diminished demyelination of the spinal cord.^490^ In addition, the authors observed similar EAE outcomes in HLA-DR4-transgenic C57BL/6IAb^null^ mice (MHC-II knockout mice expressing a transgenic hybrid MHC-II molecule comprising the peptide-binding domain of human HLA-DR4 and the membrane-proximal domain of mouse IE (DR4-IE)) that were treated once clinical symptoms had already been established. These mice were immunized with human (h) PLP_175–192_ or hMOG_97–108_ peptides and subsequently treated with hPLP_175–192_/DR4-IE or hMOG_97–108_/DR4-IE nanoparticles after the development of clinical symptoms.^490^ Moreover, to delve deeper into the specificity of pMHC-loaded nanoparticle therapy, the same authors induced EAE in C57BL/6IAb^null^ HLA-DR4-IE-transgenic mice by immunizing them with hPLP_175–192_. Subsequently, they treated diseased mice with various interventions: hPLP_175–192_/DR4-IE nanoparticles (as a positive control), uncoated nanoparticles (as a negative control), EAE-relevant hMOG_97–108_/DR4-IE nanoparticles (presenting a peptide distinct from the one used to induce the disease), or mCII_259–273_/DR4-IE nanoparticles (relevant to collagen-induced arthritis). While mCII_259–273_/DR4-IE nanoparticles exhibited no therapeutic efficacy, hMOG_97–108_/DR4-IE nanoparticles effectively mitigated EAE to a level comparable to that of the positive control.^490^ Therefore, pMHC-based nanomedicines represent a new class of therapeutics for autoimmune diseases that are capable of resolving complex autoimmune responses in a disease- and organ-specific manner without compromising systemic immunity.

##### Peptide conjugated to antibodies

Another subject of interest regarding the induction of immune tolerance in MS involves the conjugation of antigens with antibodies specific for APC receptors, such as the receptor DEC205, which is specifically expressed on DCs and thymic epithelial cells and is involved in antigen presentation. This approach is grounded in the hypothesis that administering antigens coupled with antibodies targeting DEC205 stimulates antigen presentation by immature DCs, subsequently initiating the differentiation of naive T cells to Treg cells in the periphery.^491^ In this context, a group of researchers aimed to target DCs with anti-DEC205 antibodies conjugated with PLP_139–151_ in the PLP_139–151_-induced SJL EAE model. These compounds strongly alleviated the clinical symptoms of the treated mice. Moreover, the investigators isolated splenocytes from treated mice and observed an anergic effect on PLP_139–151_, accompanied by a significant reduction in IL-17 secretion.^492^ Another study in which DCs were targeted with anti-DEC205 antibodies fused to MOG_35–55_ indicated that EAE was ameliorated in a MOG_35–55_ mouse model through the generation of suppressive FoxP3^+^ Treg cells.^493^

Antibodies against DC inhibitory receptor 2 (DCIR2), which specifically target CD11c^+^CD8^−^ DCs, have also been tested in EAE. Anti-DCIR2 conjugated to PLP_139–151_ was administered to SJL EAE mice immunized with PLP_139–151_. This approach significantly improved EAE symptoms.^494^ The effect seems to be related to a reduction in IL-17- and IFN-γ-producing pathogenic T cells together with an expansion of Treg cells. The investigators also concluded that treatment with DCIR2^+^ fusion antibodies prompted the antigen-specific activation and proliferative expansion of natural Treg cells derived from the thymus.^494^

In a recent preclinical study, researchers aimed to prevent and suppress established EAE in mice by inducing T-apoptosis via a combination of anti-CD4 and anti-CD8 antibodies alongside the administration of MOG_35–55_ or PLP_139–151_ in MOG_35–55_- or PLP_139–151_-induced EAE. This strategy led to the generation of antigen-specific Treg cells and demonstrated efficacy in both preventive and therapeutic approaches. Mechanistic investigations revealed that apoptotic T cells induced by antibodies stimulate macrophages to produce TGF-β. This, combined with the administration of autoantigenic peptides, facilitated the generation of antigen-specific Treg cells.^495^ However, the authors of the study acknowledged a limitation concerning the uncertain safety of combined CD4^+^ and CD8^+^ T-cell depletion.^495^

Considering the above findings, peptide-loaded carrier approaches enhance antigen delivery, promote long-lasting immune tolerance, and minimize systemic side effects, making them a promising strategy for MS treatment. Their versatility in carrier selection allows for targeted modulation of multiple autoimmune pathways, offering advantages over traditional immunotherapies.^496^ However, obstacles such as high production costs, manufacturing complexity, potential immune activation, and regulatory hurdles limit clinical adoption. To realize their full potential, optimized formulations, precise dosage control, and advanced delivery systems are needed. Robust clinical validation is essential to establish long-term efficacy and safety. Overcoming these barriers will be critical in translating promising preclinical findings into viable antigen-specific therapies for MS.

### Cellular immunotherapy approaches

Antigen-specific cell-based tolerogenic therapies represent intriguing approaches for restoring self-tolerance by specifically targeting and diminishing autoreactive T-cell clones.^497,498^ The primary cellular immunotherapies employed in the context of MS include T-cell vaccination, tolerogenic DCs and antigen-specific chimeric antigen receptor-modified Treg cells. These will be described further below (Fig. 5e).

#### T-cell vaccination

The conventional method of T-cell vaccination (TCV) has spurred an alternative strategy aimed at inducing T-cell-dependent inhibition of autoimmune responses via immunization with attenuated autoreactive T cells.^499^ The fundamental principle of the TCV strategy is to initiate APC processing of self-components derived from the attenuated autoreactive-T-cell population. This process yields a broad spectrum of T-cell specificities that potentially modulate or eliminate pathogenic autoreactive T cells through two recognized regulatory mechanisms: antidiotypic and anti-ergotypic responses.^499^ In succinct terms, the anti-idiotypic or anti-clonotypic response is directed toward antigenic determinants (peptides) derived from the TCRs of autoreactive CD4^+^ or CD8^+^ T cells. Conversely, the anti-ergotypic response targets antigenic determinants originating from activation markers (ergotope molecules) of T cells.^500^ In summary, the immunization protocol utilizing attenuated autoreactive T cells involves a multifaceted cellular reaction directed at autoreactive T cells. In addition to anti-idiotypic and anti-ergotypic T cells, less commonly occurring lymphocyte populations, such as gamma delta (γδ) T cells and natural killer (NK) cells, also expand upon exposure to the vaccine. This observation suggests potential roles for these cells in the regulation of T–T-cell interactions within the immune system.^500^

In an initial preclinical study, some authors revealed that intravenous inoculation of syngeneic rats with an attenuated MBP-reactive T-cell line conferred protection to naive rats against the subsequent induction of EAE.^501^ Furthermore, in another study utilizing an EAE adoptive transfer model, T-cell vaccination prevented the development of adoptively transferred EAE in Lewis rats following subsequent challenge with MBP in complete Freund’s adjuvant (CFA). However, although T-cell vaccination prevents the development of effector cells involved in EAE immunopathogenesis, it does not inhibit the initial response of MBP precursor cells or prevent the formation of MBP memory cells.^502^

With respect to the unclear TCV mechanism, another study examined the role of the CD28:CD80/86 interaction in TCV-induced protection in a murine model of EAE. They reported that autoreactive Th1 cells exhibit increased expression of both CD80 and CD86 compared with Th2 cells. Blockade of CD80/86 on the surface of the vaccinated T cells reduced the inhibition of antigen-specific CD4^+^ and CD8^+^ T-cell proliferation. These findings suggest that the expression of CD80/86 on autoreactive T cells is essential for their recognition by the immune system and the resulting protection against EAE in mice subjected to TCV.^503^ In another study, to clarify the mechanism underlying TCV, the authors employed anti-MBP encephalitogenic T cells engineered to express green fluorescent protein (GFP). In naive or control-vaccinated rats injected with anti-MBP encephalitogenic T cells, high levels of those effector T cells along with macrophages, CD8^+^ T cells, and non-GFP CD4^+^ T cells were detected in the spleen, parathymic lymph nodes, and spinal cord. In contrast, TCV-treated rats presented minimal encephalitogenic T cells during disease onset and reduced numbers of macrophages and CD4^+^ and CD8^+^ T cells in the spinal cord. Splenocytes from the control groups secreted IFN-γ in response to MBP and presented high numbers of IFN-γ-secreting CD4^+^ and CD8^+^ T cells in the spinal cord at the peak of the disease. In the TCV-protected groups, splenocytes did not react to MBP but secreted IFN-γ in response to irradiated encephalitogenic T cells, indicating an antidiotypic response. Therefore, TCV significantly reduces effector T-cell numbers in the CNS and lymphoid organs, diminishes Th1 cytokine-producing cells in the CNS, and induces the emergence of T cells responsive to anti-MBP effector T cells.^504^

These favorable outcomes observed through the TCV approach in animal models of MS prompted subsequent exploration in clinical trials.^502–504^ An initial pilot trial encompassing eight patients diagnosed with RRMS or SPMS was conducted, where patients received TCV with irradiated T cells reactive to MBP. Among the treated patients with RRMS, there was a noteworthy decrease in the frequency of exacerbations in the two-year period of follow-up after TCV compared with the two-year period prior to treatment. Notably, patients who underwent treatment exhibited a comparatively smaller increase in the size of brain lesions than untreated patients did.^505^ However, it is essential to acknowledge that in three out of eight patients, lesions and relapses exhibited exacerbation subsequent to vaccination.^505^ From a mechanistic standpoint, TCV elicits an antidiotypic T-cell response, leading to a gradual depletion of MBP-reactive T cells in all patients.^506^ Two more trials involving MS patients (49 and 54 patients, respectively) explored immune responses from vaccination with MBP-reactive T cells. The results indicated that reduced MBP-reactive T cells were correlated with delayed MS progression in both RRMS and SPMS patients.^507,508^ Another trial with four SPMS patients used myelin-reactive T-cell lines and revealed that two patients maintained a stable EDSS score, one patient demonstrated an improvement of one EDSS step, and the remaining patient experienced progression in the EDSS score. Following the second inoculation, there was a continuous reduction in the number of circulating myelin-reactive T cells, which specifically targeted the peptides MBP_143–168_, PLP_104–117_, and MOG_43–55_. Furthermore, TCV led to a decrease in myelin-specific IL-2- and IFN-γ-secreting T cells.^509^ Taken together, these findings illustrate the viability and safety of the procedure while also offering some evidence of its clinical effectiveness.

The promising results in terms of feasibility, safety, and immune response associated with this approach have prompted the initiation of a randomized phase IIb clinical trial to evaluate the potential effectiveness of TCV with tovaxin, an in vitro expanded myelin-reactive T cells manufactured against up to six immunodominant peptides derived from MBP, MOG and PLP. The trial was conducted in 150 subjects to evaluate safety and efficacy in RRMS and CIS patients. In this sense, tovaxin has a positive safety profile.^510^ Although the primary analysis did not reveal significant clinical or radiological advantages of tovaxin immunotherapy, a more detailed examination of individuals with heightened disease activity indicated improvements in the annual relapse rate and disability progression. Nevertheless, the clinical benefits observed were not accompanied by treatment-related enhancements in MRI measurements.^510^

To conclude, the promising results derived from a variety of clinical trials undertaken across various phases of MS provide a rationale for considering TCV as a feasible alternative to MS treatment. Despite the limitations faced by particular trials, both the approach of vaccinating with T-cell clones and the utilization of multiple antimyelin cell lines (polyclonal vaccines) demonstrate the feasibility, safety, and clinical efficacy inherent in TCV for MS. Nonetheless, further investigations encompassing more extensive patient cohorts and prolonged periods of follow-up are needed to increase the robustness and validity of these observations.

#### Tolerogenic dendritic cell-based treatment

DCs play a vital role in both activating adaptive immune responses to eliminate invading pathogens and establishing tolerance to self-components for the maintenance of immunological and tissue homeostasis.^511,512^ Moreover, the documented ability of mature DCs to transition conveniently into tolerogenic DCs (tolDCs) augments their potential to elicit specific self-tolerance.^202^ The concept of tolDCs serving as initiators of immune tolerance is based on their characterized immature or semimature phenotype, which is characterized by low expression of costimulatory molecules (CD80, CD83, and CD86) and MHC molecules, along with altered cytokine production featuring heightened regulatory cytokines such as IL-10 and TGF-β, alongside reduced expression of proinflammatory cytokines.^202^ This microenvironment contributes to the induction of T-cell anergy and the promotion of Treg cell generation.^202^ In this context, several endeavors have been directed at differentiating autologous PBMCs or bone marrow-derived cells (BMDCs) into tolDCs via the use of pharmacological agents or immunomodulatory cytokines in the presence of autoantigens.^425^ One of the most noteworthy successes was achieved by generating autologous tolDCs from monocyte-derived cells cultured in the presence of 1α,25-dihydroxyvitamin D3 (VitD3) in combination with myelin peptides.^513,514^ The administration of VitD3-tolDCs pulsed with MOG_40–55_ to EAE-induced mice induced antigen-specific T-cell tolerance, resulting in a decreased incidence of disease development when the mice were treated preventively and a reduction in disease severity when MOG-pulsed VitD3-tolDCs were administered once clinical signs were already established.^515^ However, it is worth noting that sustained clinical benefits in EAE necessitate repeated administration of tolDCs.^516^

In the context of clinical trials, a phase 1b study included eight patients with MS and four patients with neuromyelitis optica spectrum disorders (NMOSD), a chronic brain and spinal cord disorder primarily associated with aquaporin-4 (AQP4) autoantigens that target up to 80% of cases (all patients in this study had anti-AQP4 antibodies as an enrollment criterion). This study investigated escalating concentrations of autologous tolDCs pulsed with peptides derived from diverse myelin proteins (such as MOG_1–20_, MOG_35–55_, MBP_13–32_, MBP_83–99_, MBP_111–129_, MBP_146–170_, and PLP_139–154_) and AQP4. The primary endpoints were focused on evaluating treatment safety and tolerability, whereas the secondary endpoints encompassed clinical and radiological outcomes.^517^ Clinically, patients’ relapse occurrence, disability, and imaging data remained stable. The authors concluded that intravenous administration of tolDCs coupled with antigen peptides proved safe and efficacious in increasing IL-10 production and the frequency of Treg cells. Furthermore, this study underscored the pivotal role of IL-10 in antigen-specific tolerance.^517^ These findings prompted the conduction of two phase I studies, which employed a ‘best of five’ design, to compare intradermal and intranodal cell transfer of incremental doses of peptide-loaded autologous tolDCs. The objective of this study was to assess safety, clinical feasibility, and immunological alterations in untreated RRMS and SPMS patients.^518^ Each patient received six repetitive administrations of 5, 10 or 15 × 10^6^ autologous tolDCs pulsed with a combination of myelin peptides via intradermal or intranodal administration, four injections, once every two weeks, and two injections, once every four weeks. The results of these studies have not yet been published (NCT02618902 and NCT02903537). Recently, tolDCs were electroporated with mRNAs encoding multiple naturally processed MOG antigens to facilitate their presentation, thus addressing the constraint posed by the limited availability of specific HLA-restricted epitopes.^519^ One study evaluated the efficacy of MOG mRNA-electroporated DCs in EAE mice and reported significant stabilization of the EAE clinical score.^519^

In summary, advancements within the scientific domains of recombinant protein expression, genome editing, and nanotechnology-driven drug delivery mechanisms, coupled with refined immunization protocols, have the potential to increase the prospective utility of tolDC vaccination in future periods.^202^

#### Antigen-specific chimeric antigen receptor-modified Treg cell therapy

An appealing therapeutic approach for MS involves the use of engineered Treg cells expressing chimeric antigen receptors (CARs) specific for myelin. This strategy addresses key limitations of polyclonal Treg cell therapy, such as the need for large cell numbers and the risk of nonspecific immunosuppression, including viral reactivation. CAR-Treg cells offer a more targeted approach by recognizing specific myelin antigens, allowing for localized suppression of autoimmune responses with fewer administered cells. CARs are composed of a single-chain variable fragment (scFv), an extracellular hinge, a transmembrane region, and intracellular signaling domains. This approach has been successfully extended to Treg cell therapies, resulting in the development of CAR-Treg cells.^520,521^ In a preclinical study, CD4^+^ T cells were engineered to express a CAR that specifically targeted MOG together with murine FoxP3 to facilitate Treg lineage commitment. Upon intranasal administration, these engineered CAR-Treg cells efficiently migrated to CNS regions, reduced clinical disease scores, and lowered the mRNA levels of proinflammatory cytokines such as IL-12 and IFN-γ in brain tissue. Notably, mice treated with MOG-specific CAR-Treg cells were protected against subsequent EAE onset, suggesting long-term immune tolerance induction.^522^

Further studies demonstrated that both MBP- and MOG-specific CAR-Treg cells significantly reduced disease progression in EAE models, whereas polyclonal Treg cells had limited efficacy. This finding reinforces the superior therapeutic potential of antigen-specific CAR-Tregs over their polyclonal counterparts, particularly in terms of antigen-targeted action, clinical efficacy, and reduction in systemic immunosuppression.^523^ The authors proposed that future studies should explore whether these CAR constructs function via direct antigen engagement or through a bystander effect within inflamed CNS tissues.^524^

It is important to distinguish this CAR-Treg cell approach from other antigen-specific Treg expansion strategies, such as those utilizing APCs. As previously discussed in the context of tolDCs, APCs can be used to induce and expand antigen-specific Treg cells by presenting relevant myelin antigens in an immunomodulatory manner.^525^ Notably, one alternative to CAR-based engineering is the ex vivo expansion of recipient-derived Treg cells stimulated by donor APCs loaded with myelin antigens, which promotes antigen-specific tolerance without the need for genetic modification.^525^ While both strategies aim to enhance Treg-mediated immune regulation, CAR-Treg cells offer a synthetic and potentially more controlled method for directly targeting and modulating autoreactive immune responses. In contrast, APC-based expansion relies on natural antigen presentation and may have broader effects depending on the tolerogenic properties of the APCs used. Recent research has increasingly focused on the unique therapeutic advantages and challenges of CAR-Treg cells, particularly in the context of autoimmune disease. In their 2022 review, Arjomandnejad et al. explored the application of CAR-Treg therapy in autoimmune diseases, highlighting its advantages over polyclonal Treg approaches in terms of specificity, efficacy, and safety.^526^ CAR-Tregs provide antigen-specific immunosuppression, enabling precise targeting of autoreactive responses while preserving global immune function, an essential feature for treating autoimmunity.^523^ Unlike polyclonal Tregs, which act broadly and may cause off-target effects, CAR-Tregs can be engineered to home to inflamed tissues and suppress immune activation in an antigen-restricted manner.^527^ This targeted nature of CAR-Tregs offers improved safety by minimizing systemic immunosuppression, a common limitation of polyclonal Treg therapies.^528^ However, the authors underscore several challenges that must be addressed for successful clinical translation. CAR-Treg production involves complex ex vivo manipulation, genetic engineering, and expansion under stringent GMP conditions, making it more resource intensive than conventional Treg manufacturing is.^527,528^ Additionally, maintaining phenotypic stability remains a critical safety concern, as loss of FOXP3 expression could lead to the emergence of proinflammatory effector cells.^529^ Importantly, the design of CAR constructs—including the choice of costimulatory domains—has been shown to modulate Treg function, with CD28-based CARs better preserving suppressive capacity than 4-1BB domains do.^530^ Despite these hurdles, advancements in gene editing technologies and automated manufacturing platforms may help overcome current limitations, reinforcing the therapeutic potential of CAR-Tregs for restoring immune tolerance in patients with autoimmune disorders.^528^

These findings suggest that antigen-specific CAR-Treg cell therapies could provide MS patients with a novel means of preventing relapses and limiting disability progression. Further research is warranted to refine CAR designs, optimize delivery strategies, and evaluate long-term safety in clinical settings.

In summary, cellular immunotherapy approaches in MS provide targeted immune modulation through strategies such as TCV, tolDCs, and CAR-Treg cells, aiming to suppress autoreactive T cells while preserving systemic immunity. These therapies demonstrate strong preclinical efficacy in EAE models, showing reduced CNS inflammation, Treg cell induction, and multiepitope targeting to mitigate epitope spreading. Unlike broad immunosuppression, cellular therapies offer a more precise approach, potentially improving treatment outcomes with fewer side effects.^531^ Early clinical trials indicate safety and modest benefits, including reduced relapse rates with TCV and increased Treg cell activity with tolDCs. However, clinical translation remains challenging owing to mixed outcomes in human trials, manufacturing complexity (e.g., autologous cell therapies, CAR-Treg cell engineering), and uncertainties regarding the durability of tolerance and immune regulation. Additional barriers such as scalability, route-dependent delivery, and long-term safety concerns (e.g., genetic modification risks) further limit their widespread adoption. Despite these limitations, cellular immunotherapies hold immense potential as personalized, antigen-specific treatments for MS. Their ability to reprogram immune responses through TCV, tolDCs, and CAR-Treg cells lays the foundation for next-generation immunotherapies. To achieve clinical viability, further research is needed to refine patient stratification, increase production efficiency, and establish long-term safety and efficacy. Overcoming these obstacles will be crucial for translating cellular immunotherapies into accessible and effective treatments for MS.^532^

#### Challenges and future directions of antigen-specific immunotherapies

Antigen-specific immunotherapies for MS are promising for eliminating the detrimental autoimmune response, stopping disease progression and sidestepping the side effects associated with currently available therapies. In the context of MS, the exact primary target antigen remains uncertain, but there is a consensus that proteins found in the myelin sheath, such as MBP, MOG, and PLP, are essential in the autoimmune response.^533^ Additionally, it is conceivable that immune responses against myelin antigens undergo fluctuations over time^534,535^. Consequently, one may reasonably postulate that the efficacy of antigen-specific therapies will depend on two critical factors: a comprehensive understanding of the precise target antigens and the ability to inhibit epitope spreading. Within this framework, antigen-specific immunotherapies should aim to regulate activated autoreactive T cells while preventing the activation of resting autoreactive T-cell clones^452^. Throughout the years, numerous strategies have been developed to induce immune tolerance in the context of MS. Among these strategies, some have successfully progressed to clinical translation, whereas others have not, often owing to nonscientific factors such as a lack of resources or insufficient intellectual property protection. However, even when these barriers are overcome, translating antigen-specific immunotherapies from preclinical models to clinical application in MS remains a considerable challenge despite promising experimental outcomes. Numerous obstacles hinder this transition, ranging from biological and mechanistic limitations to technical, regulatory, and economic barriers. Addressing these obstacles is crucial for the successful implementation of these therapies. Table 4 summarizes the key challenges and potential strategies to overcome them, highlighting critical areas for future research and development. One of the primary difficulties lies in the heterogeneity of MS pathogenesis. While preclinical studies often focus on a single antigen, such as MOG, MBP, or PLP, MS involves dynamic antigen spreading, necessitating multiepitope strategies to achieve broad and durable immune tolerance. Moreover, the experimental EAE model does not fully replicate the complexity of human MS. Many interventions that demonstrate efficacy in EAE fail in clinical trials because of differences in immune system regulation, antigen processing, and HLA-dependent antigen presentation^423,428,505^ Therefore, enhancing preclinical models to better mimic human immune responses may provide more predictive insights into clinical efficacy.

**Table 4:** Challenges and strategic solutions for advancing antigen-specific immunotherapies in multiple sclerosis

Challenges	Proposed solution
Uncertainty in target antigens	Develop multiepitope strategies to account for antigenic variability and immune response fluctuations
Epitope spreading	Use combination therapies targeting multiple myelin antigens to prevent immune escape
Preclinical models’ limitations	Improve humanized mouse models and integrate human antigen analogs to enhance clinical translatability
Lack of sustained immune tolerance	Focus on long-term immune monitoring and explore adjuvant therapies that enhance tolerance maintenance
Unintended immune activation	Modify vaccine adjuvants and optimize nanoparticle formulations to reduce pro-inflammatory risks
High production and scalability issues	Optimize production pipelines, automate cell culture systems, and explore cost-effective synthetic formulations
Optimal route of administration	Prioritize intravenous delivery to better target immune-regulatory organs like the spleen and liver
Lack of reliable biomarkers for patient stratification	Identify and validate predictive biomarkers to optimize patient selection and improve clinical trial success rates
Regulatory and approval barriers	Develop adaptive clinical trial designs and engage early with regulatory agencies to streamline approval pathways
Variability in clinical trial outcomes	Implement stratified trial designs based on HLA typing and immune profiling to enhance treatment personalization

*HLA* human leukocyte antigen

In this context, the creation of “humanized” mouse models for autoimmune diseases presents a promising avenue to address these challenges, as it allows for preclinical assessment of compounds specifically designed for human targets. Moreover, the substitution of murine autoantigens with their human analogs, aimed at overcoming species-specific variations in primary structure, may facilitate the clinical translation of antigen-specific therapies^536^. Another fundamental limitation is the lack of sustained immune tolerance. While many antigen-specific immunotherapies demonstrate transient efficacy, their long-term immunomodulatory effects remain uncertain. This issue is exacerbated by the tendency of clinical trials to prioritize short-term endpoints, such as MRI lesion reduction, rather than durable disease remission. Furthermore, unintended immune activation presents a critical risk, particularly with certain delivery platforms. For example, DNA vaccines containing CpG motifs can trigger innate immune pathways, leading to proinflammatory responses that counteract tolerance induction. Peptide-based approaches, while highly specific, often suffer from poor stability and bioavailability and require advanced delivery mechanisms to increase their efficacy^537^. Advances in biomaterials and nanotechnology also offer potential solutions, with PEGylation^538^, lipid nanoparticle encapsulation^539^ and sustained-release carriers improving antigen stability and bioavailability.^465^

Technical and manufacturing constraints further complicate clinical translation. Peptide instability and rapid degradation reduce therapeutic efficacy, whereas DNA-based therapies often exhibit low transfection efficiency and variable antigen expression. Cellular therapies, including tolDCs and CAR-Treg cells, introduce additional complexity owing to their reliance on autologous cell collection, ex vivo expansion, and reinfusion. The need for highly personalized manufacturing not only increases costs but also limits scalability, making widespread application impractical under current conditions. The high production costs associated with nanoparticle-based vaccines, DNA plasmids, and cell-based therapies also pose considerable financial challenges. Unlike small-molecule drugs, which benefit from established large-scale manufacturing pipelines, antigen-specific immunotherapies require specialized production facilities, significantly increasing costs and limiting their commercial viability.^540–543^

Additionally, the route of administration plays a crucial role in determining therapeutic success.

The route of antigen administration represents another important challenge for tolerization approaches. This encompasses different modalities, including oral, nasal (typically mucosal), transdermal, intravenous, intramuscular or intranodal delivery of cells or other carriers, i.e., nanoparticles coupled to autoantigens. Oral administration has long been regarded as the preferred method for inducing tolerance, primarily because of the significant role played by the mucosal immune system in ensuring tolerance to food antigens.^384^ However, clinical trials investigating oral tolerance have not yet demonstrated its effectiveness in treating MS. Additionally, when cells or nanoparticles containing autoantigens are administered, there is a risk of eliciting proinflammatory responses, which must be considered when designing a strategy of this nature to induce tolerance.^424^ In recent antigen-specific approaches, emphasis has been placed on the importance of the spleen and the liver, as they are pivotal in preserving immune balance. Therefore, the intravenous delivery of antigens, peptide-linked cells, or nanoparticles has been considered the most efficacious strategy for specifically addressing these organs, and it is generally considered a safe method. Therefore, the location where tolerizing peptides undergo degradation and the environment in which they are presented to immune cells are important features to consider.^384^ In addition, antigen-specific immunotherapies leveraging cell carriers and nanoparticles employ sophisticated targeted delivery mechanisms that transport specific cargo to predetermined destinations within the body. For this reason, these carriers should be meticulously engineered with surface modifications serving as intricate “guidance systems”, facilitating binding to receptors on target cells or tissues and thereby ensuring precise and directed delivery. Adjusting the charge, size, and even the shape could help the carrier evade clearance by immune cells and navigate toward the target site more efficiently. Moreover, guaranteeing antigen discharge in close proximity to relevant immune cells and a controlled release of cargo would maximize interaction and elicit a focused immune response.^544^

Further challenges confront antigen-specific approaches. These challenges include decisions related to dosage, administration frequency, selection of antigens, and even the composition of combinatory therapies. The latter should ideally include a potent anti-inflammatory treatment to reduce disease activity and create an optimal environment for tolerance induction via an antigen-specific approach. This is particularly crucial, as antigen-specific therapy should ideally be initiated during the early stages of the disease, when inflammatory activity is prominent. Specifically, in the case of MS patients with high disease activity, it has been proposed to explore the duration of combinatory therapy before transitioning to a monotherapy approach using a tolerizing agent.^384^

Beyond these technical hurdles, regulatory and economic barriers further restrict clinical progress. One of the most pressing concerns is the absence of reliable biomarkers for patient stratification. Unlike conventional immunosuppressants, which act broadly, antigen-specific immunotherapies require precise patient selection to maximize efficacy. Without validated biomarkers, clinical trials encompass heterogeneous patient populations, potentially diluting therapeutic effects and leading to inconclusive results. Consequently, it becomes imperative to develop biomarkers for the following purposes: guiding the determination of appropriate dosages, assessing the induction of immune tolerance, gauging the duration of the tolerization effect alongside clinical outcome parameters, and distinguishing between responder and nonresponder subjects. Furthermore, it is recommended that clinical trial designs incorporate mechanistic investigations and define the optimal target population most likely to respond positively to the treatment.^545^

Moreover, regulatory agencies impose rigorous safety requirements, particularly for novel approaches such as nanoparticle-based formulations and CAR-Treg cells. These stringent standards, coupled with the need for long-term safety assessments, prolong approval timelines and deter investment in antigen-specific immunotherapy development.

Lessons from clinical trials further underscore the complexity of antigen-specific immunotherapy translation. Peptide-based therapies, despite demonstrating high specificity, exhibit considerable variability in patient response, largely owing to differences in HLA type and immune reactivity. For example, MBP_82–98_ showed promise in early-stage trials but failed in phase III trials, highlighting the necessity for patient stratification.^398^ DNA vaccination strategies, such as BHT-3009, have confirmed safety and tolerability but have yet to demonstrate substantial long-term efficacy.^546^ While these vaccines induce broad immune tolerance, MRI-based improvements have been inconsistent, suggesting that sustained antigen exposure or combinatorial approaches may be required to achieve meaningful clinical outcomes. Peptide-loaded nanoparticles, although promising in EAE models, have faced slow clinical translation due to safety concerns and regulatory uncertainty surrounding nanomedicine. Optimizing nanoparticle formulations and addressing long-term biocompatibility concerns will be critical for advancing these approaches. Cellular immunotherapy, including tolDCs and TCV, represents a highly precise treatment modality but remains hindered by complex manufacturing requirements and variability in patient response. CAR-Treg cells, while conceptually powerful, face significant regulatory and scalability challenges that must be addressed before widespread implementation can be achieved.

Ultimately, antigen-specific immunotherapies offer a transformative approach to MS treatment by providing antigen-specific immune modulation with reduced systemic immunosuppression. However, their clinical viability hinges on overcoming the numerous translational barriers outlined above. Future research must focus on refining delivery systems, optimizing patient stratification, and integrating biomarker-driven treatment approaches. Additionally, combination strategies that leverage the strengths of antigen-specific immunotherapies alongside existing immunomodulatory therapies may offer the best path forward. Addressing these challenges in a systematic manner will be essential to unlocking the full therapeutic potential of antigen-specific immunotherapies in MS.

## Conclusions

The ever-evolving epidemiology of MS, coupled with its diverse clinical phenotypes and complex pathophysiology, underscores the challenges in disease management. Our increasing understanding of MS etiology and pathogenesis has paved the way for more tailored therapeutic approaches, improving treatment precision. Despite significant advancements in DMTs, their efficacy is often accompanied by immune suppression-related risks and adverse effects, necessitating safer and more targeted alternatives.

Antigen-specific immunotherapies represent a paradigm shift in the treatment landscape of MS, offering unprecedented precision in modulating pathogenic immune responses while minimizing off-target effects associated with current immunotherapies. Beyond MS, these approaches hold considerable promise for addressing a broader spectrum of organ-specific autoimmune diseases, potentially redefining how we approach immune tolerance across diverse clinical contexts. The ultimate objective of these therapies lies in restoring immune homeostasis, paving the way for long-term disease remission and improved quality of life for patients. As advancements in biotechnology and immunology converge, the transition from experimental frameworks to clinically viable antigen-specific therapies is no longer a distant prospect but an imminent reality. The integration of these transformative strategies into MS management represents a crucial step toward fulfilling the unmet needs of this complex disease.
